# Fluorescent labeling of genomic loci in *Drosophila* imaginal discs with heterologous DNA-binding proteins

**DOI:** 10.1016/j.crmeth.2022.100175

**Published:** 2022-03-09

**Authors:** Rebecca K. Delker, Ross H. Munce, Michelle Hu, Richard S. Mann

**Affiliations:** 1 Department of Biochemistry and Molecular Biophysics, Columbia University Irving Medical Center, New York, NY, USA; 2 Department of Neuroscience, Columbia University Irving Medical Center, New York, NY, USA; 3 Department of Systems Biology, Columbia University Irving Medical Center, New York, NY, USA; 4 Department of Genetics and Development, Columbia University Irving Medical Center, New York, NY, USA; 5 Mortimer B. Zuckerman Mind Brain Behavior Institute, Columbia University, New York, NY, USA

**Keywords:** fluorescence microscopy, 3D nuclear architecture, genomic foci, LacI/LacO, ParB/ParS, gene regulation

## Abstract

Using the *Drosophila melanogaster* Hox gene *Ultrabithorax* (*Ubx*) as an example, we demonstrate the use of three heterologous DNA-binding protein systems—LacI/LacO, ParB1/ParS1, and ParB2/ParS2—to label genomic loci in imaginal discs with the insertion of a small DNA tag. We compare each system, considering the impact of labeling in genomic regions (1) inside versus outside of a transcribed gene body and (2) with varying chromatin accessibility. We demonstrate the value of this system by interrogating the relationship between gene expression level and enhancer-promoter distance, as well as inter-allelic distance at the *Ubx* locus. We find that the distance between an essential intronic *cis-*regulatory element, *anterobithorax* (*abx*), and the promoter does not vary with expression level. In contrast, inter-allelic distance correlates with *Ubx* expression level.

## Highlights

• Tools for labeling genomic loci with three fluorescent binding proteins
• Characterization of these labeling tools at distinct genomic contexts
• Inter-allelic distance at *Ubx* correlates with gene expression levels
• Enhancer-promoter distance at *Ubx* is decoupled from gene expression levels

## Motivation

Understanding how and when genomic regions interact with each other is needed to dissect genome architecture and understand gene regulation. Toward this end, it is important to have tools to efficiently label DNA elements in both live and fixed tissues. We sought to expand the toolbox available to the *Drosophila* research community and to assess the strengths and weaknesses of each tool by labeling specific DNA sequences in the nuclei of imaginal discs.

## Graphical abstract

**Figure:**
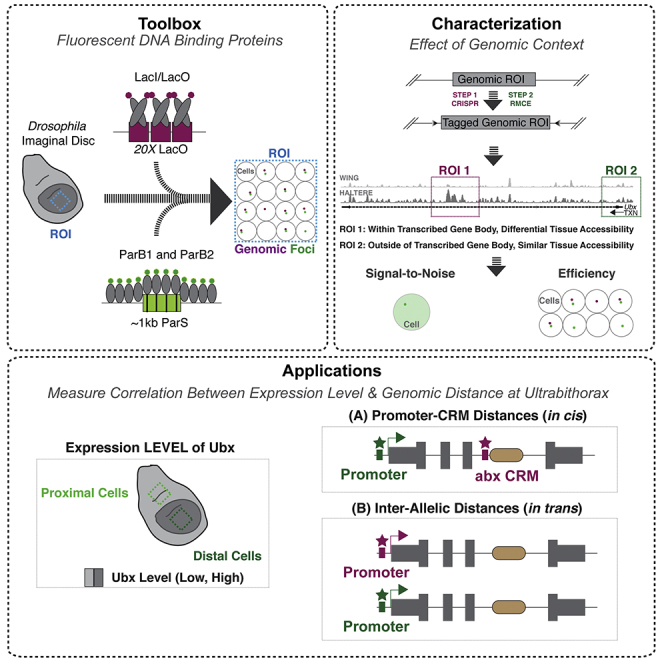

## Teaser

Labeling genomic regions of interest with heterologous fluorescent proteins (FPs) allows the localization of these regions in three dimensions within nuclei relative to each other and to additional nuclear features. Delker et al. test and characterize the use of three FPs in distinct genomic contexts of *Drosophila melanogaster* imaginal discs, as well as use two FPs to interrogate genomic proximity at the Hox gene *Ultrabithorax*.

## Introduction

An astonishing feature of eukaryotic cells is that the length of DNA is several orders of magnitude larger than the size of the nucleus in which it is packed. Yet the task of correctly transcribing the information encoded in the DNA is not compromised, and many results point to the organization of chromatin within the nucleus as being critical (Bonev and Cavalli, 2016). How this organization is established and maintained, and its relationship with gene regulation, remain important questions in the field (Maeshima et al., 2019; Bonev and Cavalli, 2016; Schoenfelder and Fraser, 2019). Thus, techniques to assay subnuclear genome organization have become increasingly important (Boettiger and Murphy, 2020; Weiss et al., 2018). This includes both genomic interactions and subnuclear gene position, particularly in relation to nuclear features, including the nuclear periphery, and key transcriptional proteins: RNA polymerase (RNAP) and transcription factors (TFs).

While both biochemical and microscopy-based techniques have been employed to interrogate subnuclear organization, we focus on the development of tools to visualize DNA. Fluorescent microscopy offers several benefits over biochemical methods: (1) single-cell resolution without cell sorting; (2) co-visualization of multiple cellular components, including protein, DNA, and RNA; and (3) live imaging. *In situ* hybridization (ISH) methodologies, developed in the 1960s with the use of radiolabeled oligonucleotide probes and improved in the 1980s with fluorescent probes (FISH), identify the subnuclear position of genomic regions of interest (ROIs) in fixed cells (Gall, 2016; Ruano-Gallego et al., 2019; Huber et al., 2018). Recent improvements in synthetic oligo production and labeling, coupled with advances in microscopy and sequential labeling methods, have enabled the use of FISH to probe genomic interactions in a high-throughput manner, as well as with kilobase resolution (Boettiger and Murphy, 2020; Boettiger et al., 2016; Mateo et al., 2019; Bintu et al., 2018; Beliveau et al., 2015). Despite these advances, FISH, like its biochemical counterparts, can only assay subnuclear organization in fixed cells. In addition, productive hybridization of probes requires the denaturation of DNA in a time-consuming process, and allele-specific labeling requires sequence differences between alleles (Beliveau et al., 2015).

The visualization of ROIs by targeting fluorescent fusion proteins (FPs) rather than oligonucleotide probes provides an alternative strategy that circumvents many of these problems but requires its own considerations. Imaging of fixed tissue can be accomplished with a faster and more streamlined protocol, and the binding of FPs without denaturation enables the option of live imaging of ROIs and provides allele-specificity through targeted insertion of the FP binding site. To accomplish this task, researchers have used a variety of protein systems over the years: (1) Lac Repressor/Operon (LacI/LacO) (Robinett et al., 1996; Straight et al., 1996; Chen et al., 2018b; Roukos et al., 2013), Tet Repressor/Operon (TetR/TetO) (Lucas et al., 2014; Alexander et al., 2019; Tasan et al., 2018), (3) Cumate Repressor/Operon (cuO/CymR) (Alexander et al., 2019), (4) Partition Protein B/Binding Sequence (ParB/ParS, ParB/INT, or ANCHOR) (Germier et al., 2017, 2018; Chen et al., 2017; Saad et al., 2014; Mariamé et al., 2018), and (5) CRISPR/Cas9 (Chen and Huang, 2014; Chen et al., 2018a; Wu et al., 2019; Gu et al., 2018). Derived from bacteria and applied to eukaryotic cells, each system requires the localization of multiple FPs to the ROI for efficient visualization. Multimerization is achieved by one of three mechanisms: (1) an array of repeated short binding sites, each of which recruits a dimer of FP (LacI/LacO, TetR/TetO, cuO/CymR); (2) a single binding tract (∼1 kb), which upon FP binding initiates further recruitment of FPs through protein-protein interactions (ParB/ParS); or (3) tiling of guide RNAs (gRNAs) across a stretch of native or inserted genomic sequence to recruit multiple FPs (Cas9). Each of these targeted regions, particularly when engineered into the genome, constitute the DNA tag necessary for FP binding.

Here we provide a resource for the *Drosophila* research community by establishing the utility of three heterologous FP labeling systems—LacI/LacO, ParB1/ParS1, and ParB2/ParS2—to visualize the subnuclear positions of genomic loci in third instar imaginal discs. We present the optimization of DNA tag size, FP construction including chosen fluorescent tag and linker sequence, and FP expression methodology. Furthermore, using the Hox gene, *Ultrabithorax* (*Ubx*), as a test locus, we conduct a comprehensive analysis of each system, comparing their efficacy at ROIs (1) inside versus outside of a transcribed gene body and (2) in regions of varying chromatin accessibility. While the achievable signal-to-noise ratio (SNR) of both ParB/ParS systems is greater than that of LacI/LacO, the latter is more resilient to variations in FP construction and targeted ROI.

We used these tools to interrogate the relationship between gene expression level with enhancer-promoter and inter-allelic distances at the *Ubx* locus, focusing on the essential intronic *anterobithorax* (*abx*) enhancer, which is located in an intron ∼45 kb away from the promoter (Pavlopoulos and Akam, 2011; Simon et al., 1990; Weatherbee et al., 1998; Delker et al., 2019; White and Akam, 1985; Bender et al., 1983; White and Wilcox, 1984, 1985; Lewis, 1978). We found that inter-allelic distance increases with increasing expression level. However, we found no significant difference in the three-dimensional (3D) promoter-*abx* distances in *cis* in populations of cells that vary in *Ubx* expression, suggesting that expression level is regulated by a mechanism that acts independently of this feature of genomic architecture.

## Results

### A two-step genome engineering process allows multiple ROI tagging in *Ubx*

**Figure 1:**
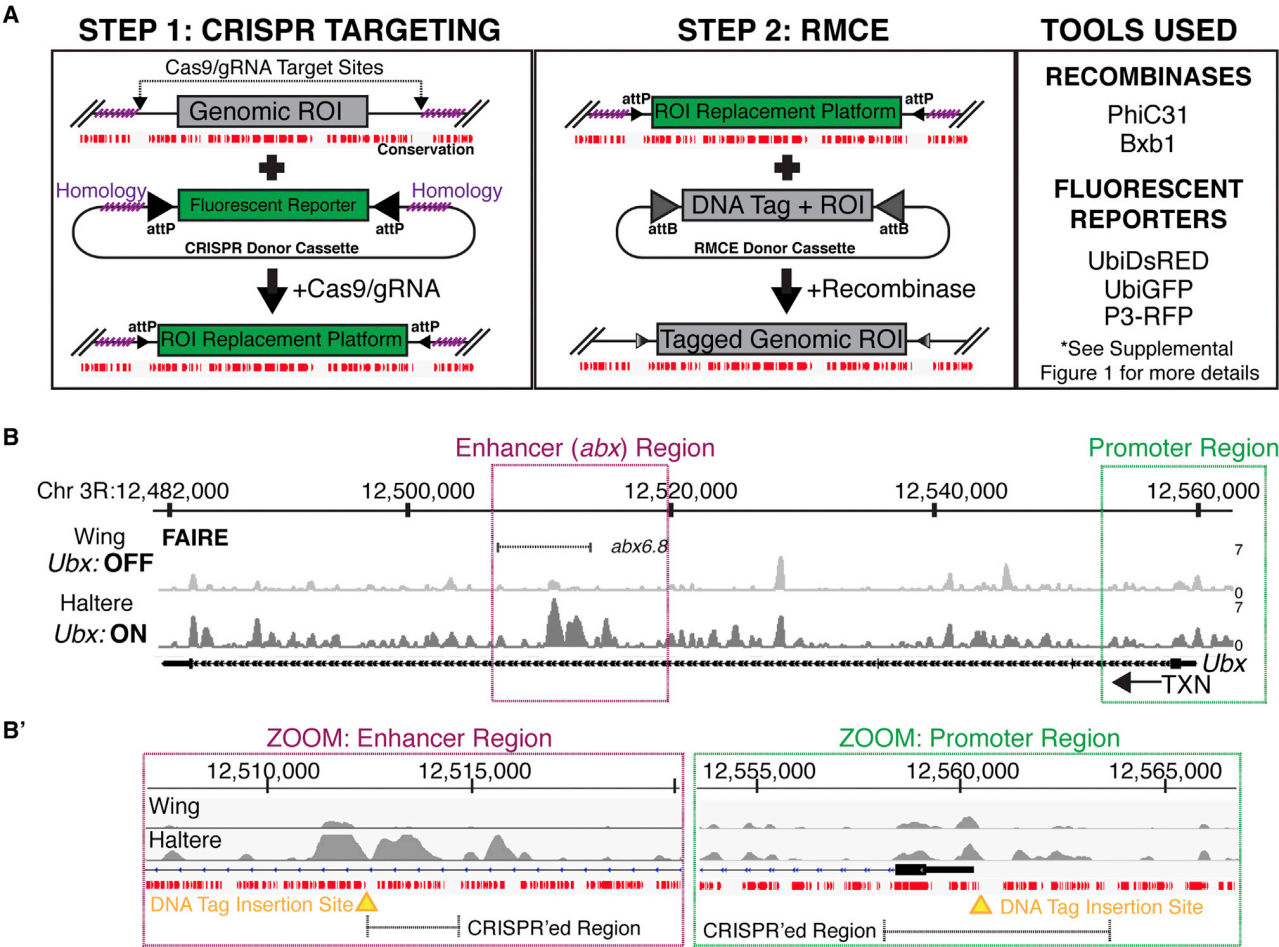
A two-step targeting strategy to tag *Ultrabithorax* at two ROIs (A) Schematic of two-step GE process. First, CRISPR replaces an ROI with a fluorescent reporter flanked by recombinase-specific attP sites. gRNAs are targeted to regions of low conservation (lack of red bars). Second, RMCE replaces fluorescent reporter with the ROI sequence plus DNA tag. Two recombinases (PhiC31, Bxb1) and three fluorescent reporters (ubiDsRed, ubiGFP, P3RFP) were used. For additional information see Figure S1. (B) Genome browser image of *Ubx* gene. Tracks show chromatin accessibility in wing and haltere discs as determined by FAIRE (McKay and Lieb, 2013). *UbxP* and *abx* are boxed in green and magenta, respectively. A 6.8 kb fragment (*abx6*.*8*) that recapitulates Ubx expression in haltere discs is shown (Simon et al., 1990). (B′) Zoom of *abx* and *UbxP*. FAIRE peaks and conservation tracks (red bars) are shown. The region replaced with CRISPR and the location of DNA tag insertion are shown. CRISPR targeting and DNA tag insertion occur in regions of low conservation.

A major barrier to using heterologous FP systems (excluding Cas9) is the requirement for targeted genome engineering (GE) to integrate the necessary ROI-proximal DNA tag (Wu et al., 2019). However, the advent of CRISPR GE has significantly eased this burden. Here, we used a two-step GE protocol that facilitates repeated tagging of an ROI. First, CRISPR targeting with a donor cassette replaces a genomic ROI with a fluorescent replacement platform compatible with subsequent recombinase-mediated cassette exchange (RMCE). In a second step, RMCE replaces the fluorescent platform with a tagged version of the ROI (Figure 1A) (Delker et al., 2019). Successful CRISPR and RMCE are easily screened through the gain and loss of fluorescent markers, respectively (Figure 1A, right). Beyond the ease of fluorescent screening, this two-step process offers additional benefits. First, once an RMCE-compatible replacement platform is generated, multiple DNA tags can be incorporated and tested without the need for additional CRISPR events, thus reducing the time and labor needed to generate new lines. We have exploited this feature throughout this study to compare the efficacy of different FP systems when targeting a DNA tag in the same position within the same ROI. Second, by making use of unique recombinase/target site combinations, replacement platforms can be built at multiple ROIs in the same fly line such that RMCE occurs independently at each ROI. Previous reports from our lab have established the efficacy of both PhiC31 and Bxb1 RMCE in *Drosophila*, and we utilize both to dual-label a single *Ubx* allele (Voutev and Mann, 2017, 2018; Delker et al., 2019). As a resource, we have included additional information in Figures 1, S1A, and S1B as well as STAR Methods.

Using our two-step GE protocol, we targeted two ROIs within the Hox gene *Ultrabithorax* (*Ubx*): the promoter (*UbxP*), and the intronic *cis*-regulatory module (CRM), *anterobithorax* (*abx*) (Figure 1B) (Simon et al., 1990; Delker et al., 2019). Because this technique is not scarless—the recombined *attP* and *attB* sequence post-recombination remains—we took measures to mitigate perturbation through insertion of the *attP* sequence into regions of low conservation (Figure 1A, absence of red bars). Expression of *Ubx* was not affected by the presence of RMCE scars as determined by the generation of mitotic clones with untagged wild-type sequence at each location (Figure S1C).

*Ubx* is notable for its role in specifying the fate of the dorsal T3 appendage, the haltere, and suppressing the fate of the dorsal T2 appendage, the wing (Delker et al., 2019; Tomoyasu, 2017; Bender et al., 1983; White and Akam, 1985; White and Wilcox, 1984, 1985). The *Ubx* locus also provides a case study to understand the efficacy of FP labeling systems under different cellular and genomic conditions. By targeting both *UbxP* (400 nt upstream of the transcription start site [TSS]) and the intronic *abx* enhancer (∼45 kb downstream of the TSS), it is possible to compare the efficacy of FP labeling outside of and within an actively transcribed gene body. Furthermore, *Ubx* expression state differs between tissues—*Ubx* is OFF in the wing and ON in the haltere—and overall chromatin accessibility at the locus differs in accordance with expression level. Compared with the OFF state of the wing, chromatin accessibility in the haltere (assessed by formaldehyde-assisted isolation of regulatory elements [FAIRE] and assay for transposase-accessible chromatin using sequencing [ATAC-seq]) is increased throughout most of the *Ubx* locus, including at *abx* (Figures 1B and 1B′) (McKay and Lieb, 2013; Loker et al., 2021). However, this is not true for the most promoter proximal accessible region where wing and haltere cells have a similar degree of accessibility despite differing in transcriptional state (Figure 1B′, right). Thus, the impact of chromatin accessibility and/or expression state on FP labeling can be analyzed. To reduce the negative potential impacts of sequence insertion, we inserted the DNA tag into non-conserved sites adjacent to a region of accessibility in haltere cells at each ROI (Figure 1B′).

### Optimization of LacI/LacO design parameters

We initially tested the two oldest FP labeling systems, TetR/TetO and LacI/LacO. Although groups working in other biological systems have successfully employed TetR FP, we were unable to detect efficient spot formation at our ROI within the intronic *abx* CRM (Tasan et al., 2018; Lucas et al., 2014; Alexander et al., 2019). Expression of a TetR-HaloTag FP targeted to a DNA tag of 48× TetO proximal to *abx* rarely induced spot formation (Figure S1D). Consequently, TetR was excluded from further analysis. While sequences used for both TetR and TetO were consistent with the literature (Figure S1D; Tables S1 and S3), the selection of the HaloTag FP could have contributed to its failure in this system.

**Figure 2:**
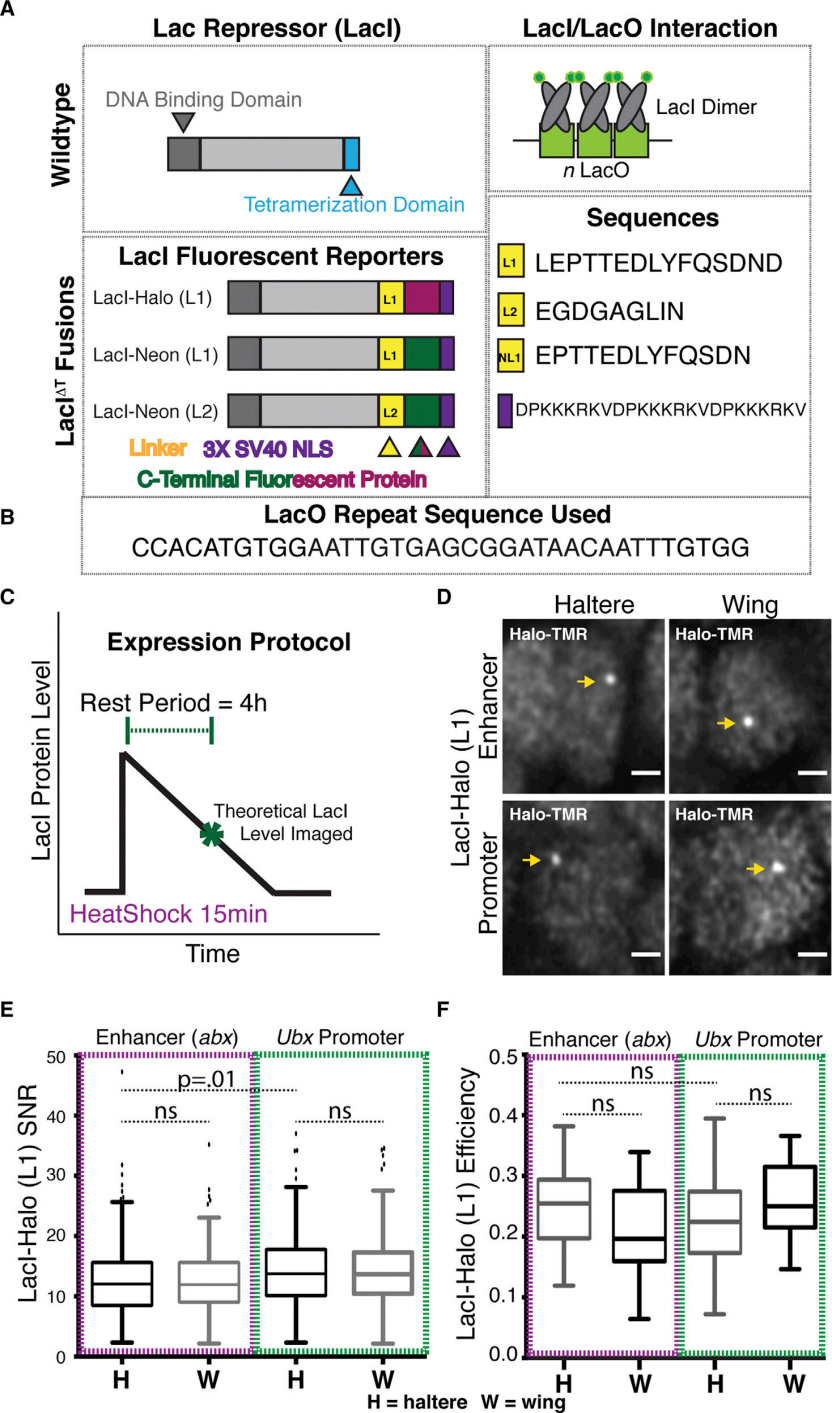
LacI/LacO labeling of genomic ROIs (A) Schematic of details of LacI-FP constructs. Wild-type LacI has an N-terminal DNA-binding domain and a C-terminal tetramerization domain. We removed the tetramerization domain and added a 3× SV40 NLS and fluorescent protein fused to LacI with a specified linker. LacI FP binds as a dimer to each LacO binding site. FPs include HaloTag and Neon. Sequences for linkers and NLS are shown. (B) Sequence of the LacO unit. (C) Protocol to express all FP fusions: a 15-min HS at 37°C followed by a 4-h rest period at 25°C prior to dissection. Theoretical protein levels following the HS and rest period are shown. (D) Examples of LacI-HaloTag (L1) foci in haltere and wing discs at each ROI. LacI-HaloTag (L1) is stained with HaloLigand TMR. Scale bars, 1 μm. (E) SNR of LacI-HaloTag (L1) stained with TMR at each ROI in wing and haltere discs. (F) Labeling efficiency of LacI-HaloTag (L1) stained with TMR at each ROI in wing and haltere discs. In (E) and (F), Tukey box plots are used. L2 is derived from Mir et al. (2018). Significance for (E) and (F) was tested using a one-way ANOVA followed by Tukey’s multiple comparisons test with *α* = 0.05 in GraphPad Prism. Reported values are adjusted p values. ns, not significant.

In contrast, LacI/LacO successfully labeled the *abx* ROI and thus was further subjected to optimization. The LacI/LacO FP labeling system is originally derived from bacteria where it plays a critical role in the gene regulatory mechanism controlling lactose metabolism. In its native context, LacI, which binds LacO as a dimer, can also tetramerize to induce the formation of a repressive loop (Brenowitz et al., 1991; Chen and Matthews, 1992). To convert LacI into a labeling tool, we removed the tetramerization domain through a short C-terminal truncation (LacI^ΔT^). Thus, for each LacO DNA tag, two LacI-FP fusions are recruited to the ROI (Figure 2A).

We tested several parameters to maximize the SNR. First, we compared the efficiency of fusing the FP to LacI at the N and C termini. Here, using the HaloTag (and a TMR-labeled Halo ligand), we establish that while both HaloTag-LacI and LacI-HaloTag can form foci at a transgene containing a 20× LacO-tagged *abx* construct, the C-terminal fusion outperforms the N-terminal fusion (Figure S2A). This likely occurs because the DNA-binding domain of LacI is N-terminal, which may be affected by an N-terminal tag (Figure 2A).

Because FP labeling systems require expression of an exogenous protein, part of the difficulty of spot detection is achieving an intensity of signal at the ROI greater than background signal from unbound FP. A number of measures can be taken to improve the SNR, including (1) adjustment of the length of DNA tag, (2) use of split FPs, and (3) the exclusion of a nuclear-localization sequence (NLS) to decrease total nuclear levels of FP. Here, we focused on optimizing both the length of DNA tag and the protocol for expression, while maintaining use of monomeric FPs and a 3× SV40 NLS for all constructs (Figure 2A). Using the heat-shock-sensitive *hsp70* promoter to drive FP expression, we are able to tune FP expression level by adjusting the length of the rest period between heat shock (HS) and analysis (Figures 2C and S2B). Following an HS of 15 min at 37°C, we tested LacI-HaloTag spot formation at *abx* 20× LacO after a rest period of 1, 2, and 4 h at 25°C. While spots were detected at each time interval, the ratio of spot intensity to background increased with increasing rest (Figure S2C). An expression protocol of 15 min HS followed by 4 h rest was used for all subsequent experiments and all FPs.

In most previous examples, the use of LacI/LacO as a labeling tool has depended on the insertion of a long tract of LacO repeats (256× LacO) amounting to ∼10 kb of inserted DNA tag and the recruitment of up to 512 LacI FPs for spot detection at an ROI (Figure S2D) (Robinett et al., 1996; Straight et al., 1996). However, increasing the number of repeats increases the risk of perturbing gene activity and decreases the accuracy of positioning the ROI within the nucleus. We thus assayed spot formation of LacI-HaloTag (N- and C-terminal) on a transgene with varying numbers of LacO repeats (256×, 160×, 64×, 40×, 20×, 10×). As expected, efficiency and SNR of labeling decreased as repeat number decreased; however, spot formation did not drop to an unusable level until 10× LacO. Thus, for all studies we use a DNA tag of 20× LacO (Figures S2D and S2E).

### LacI/LacO labeling of both *Ubx* ROIs is robust with moderate efficacy

Using the basic design parameters established above, we sought to better understand the efficacy of LacI/LacO FP labeling under different cellular and genomic contexts using *Ubx* as a model locus. We inserted a DNA tag containing 20× LacO binding sites (Figure 2B) upstream of the *UbxP* and within the intronic *abx* CRM. Using our established expression protocol (Figure 2C), we compared three C-terminally tagged LacI-FP fusions for their ability to label each ROI in cells of the distal third instar wing disc (*Ubx* OFF) and distal third instar haltere disc (*Ubx* ON). Two notable differences exist within the LacI fusions: (1) the FP used (either HaloTag-TMR [red] or Neon [green]); and (2) the linker sequence between LacI and the FP (either L1 or L2) (Figure 2A). L1 and NL1 (N-terminal linker used) are derived from commercially available N-terminal and C-terminal HaloTag constructs from Promega; L2 is derived from an mNeon-TF fusion from Mir et al. (2018) (Figures 2A and S2A). Comparisons between LacI-HaloTag (L1), LacI-Neon (L1), and LacI-Neon (L2) provide information on the relative efficacy of both the tag and the linker at each ROI (Figure S3). Here, we analyze FP efficacy with the use of two metrics. Labeling efficiency measures the ratio of nuclei with spot detection over total nuclei; and SNR is a measure of the maximum intensity of the spot relative to background FP signal (Chen et al., 2018a). Using these metrics, all LacI-FP systems can effectively label both genomic ROIs tested, though with some variability. Labeling efficiency ranged from a mean value of 0.18 to 0.30 (1.7-fold change) and SNR from a mean value of 12.68 to 26.83 (2.1-fold change) (Figure S3). Of all of the FP constructs, LacI-HaloTag (L1) displayed the greatest robustness to labeling context with minimal sacrifice to efficiency and SNR (Figure S3). No large differences exist in either efficiency or SNR in LacI-HaloTag (L1) labeling of accessible (*abx* in haltere) versus inaccessible (*abx* in wing) ROIs nor at ROIs within a transcribed gene body (*abx* in haltere) versus outside of a gene body (*UbxP* in haltere) (Figures 2D, 2E, and 2F). LacI-Neon constructs, which typically exhibit both higher efficiency and SNR compared with LacI-HaloTag in haltere samples, show greater labeling variability between tissues. This is particularly true for the efficiency of labeling at the intronic CRM, *abx*, which is significantly higher in haltere as compared with wing cells (Figure S3C). Thus, while increased efficiency can be achieved in the accessible *abx* ROI of the haltere, this gain is lost in the inaccessible *abx* ROI of the wing. Finally, a comparison of LacI-Neon constructs (L1 and L2) suggests that LacI-FP fusions are insensitive to the linker used. No significant differences exist in SNR or efficiency between LacI-Neon (L1) and LacI-Neon (L2) (Figure 4E). Thus, although minor differences exist between the different LacI-FP constructs, all can be effectively used to label all genomic ROIs. Spot detection can be performed in four channels using either a Neon FP or a HaloTag FP with commercially available fluorescent Halo ligands.

**Figure 3:**
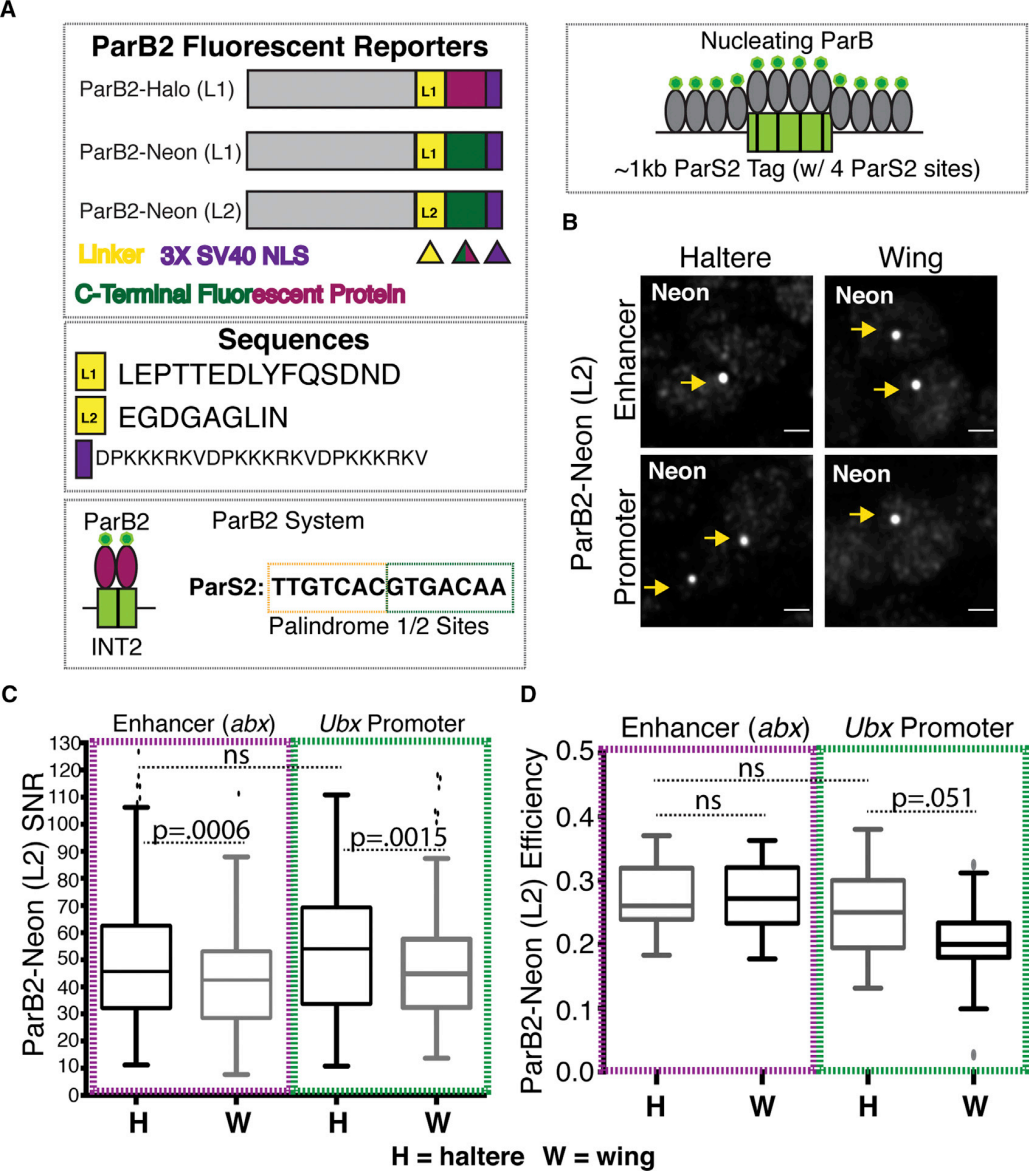
ParB2/ParS2 labeling of genomic ROIs (A) Schematic of details of ParB2 FP constructs. A 3× SV40 NLS and FP (HaloTag, Neon) is fused to the C terminus using two linkers (L1, L2). ParB2 binds ParS2 sequences and nucleates additional ParB2 with protein-protein interactions. The ParS2 binding sites from Dubarry et al. (2006) are shown, as well as sequences for the linkers and NLS. (B) Examples of ParB2-Neon(L2) spot formation at each ROI in haltere and wing discs. Scale bars, 1 μm. (C) SNR of ParB2-Neon(L2) at each ROI in haltere and wing discs. (D) Labeling efficiency of ParB2-Neon(L2) at each ROI in haltere and wing discs. In (C) and (D), Tukey box plots are used. Significance for (C) and (D) was tested using a one-way ANOVA followed by Tukey’s multiple comparisons test with *α* = 0.05 in GraphPad Prism. Reported values are adjusted p values. ns, not significant.

**Figure 4:**
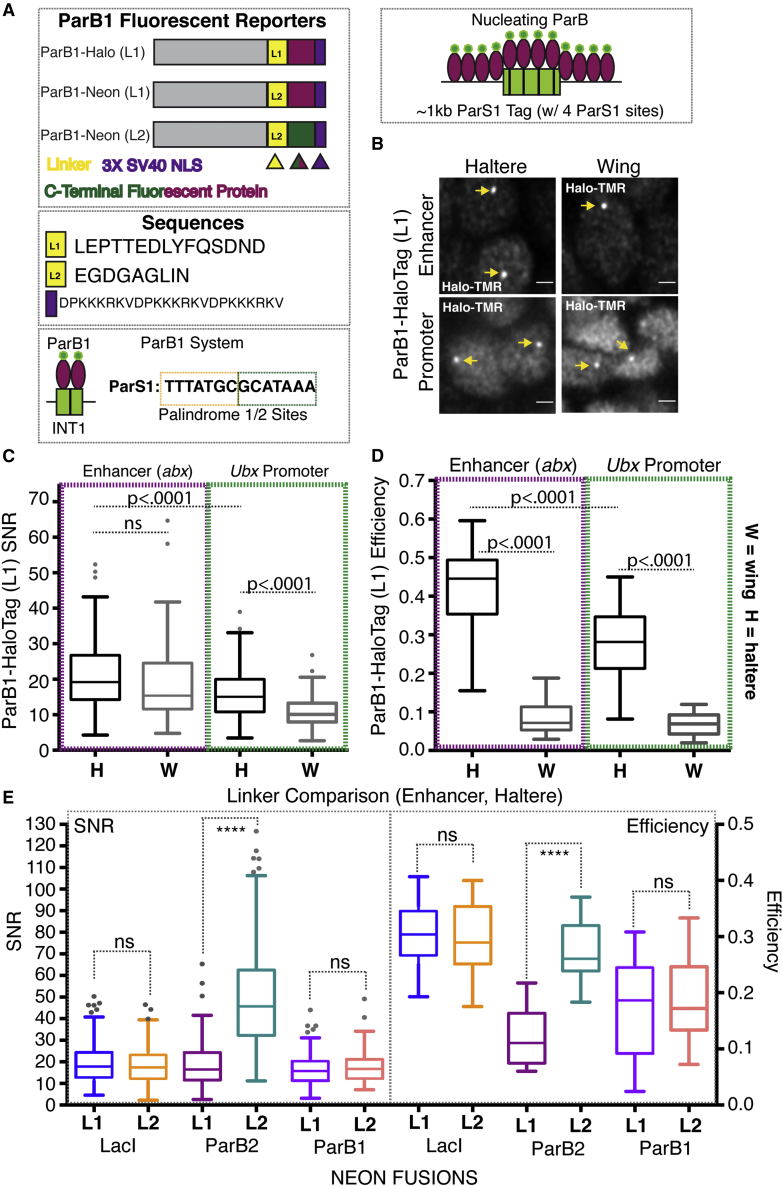
ParB1/ParS1 labeling of genomic ROIs (A) Schematic of details of ParB1 FP constructs. A 3× SV40 NLS and FP (HaloTag, Neon) is fused to the C terminus using two possible linkers (L1, L2). ParB1 binds ParS1 sequences and nucleates additional ParB1 with protein-protein interactions. The ParS1 binding sites from Dubarry et al. (2006) are shown, as well as sequences for linkers used and NLS. (B) Examples of ParB1-HaloTag (L1) spot formation at each ROI in haltere and wing discs. (C) SNR of ParB1-HaloTag (L1) at each ROI in haltere and wing discs. (D) Labeling efficiency of ParB1-HaloTag (L1) at each ROI in haltere and wing discs. (E) A comparison of SNR (left y axis) and labeling efficiency (right y axis) of each FP with L1 and L2. Tukey box plots are used in (C), (D), and (E), where one-way ANOVA followed by Tukey’s multiple comparisons test with *α* = 0.05 in GraphPad Prism was used to determine statistical significance. ∗∗∗∗p < 0.0001; ns, not significant (p > 0.05). Reported p values are adjusted p values. Scale bar is 1micron.

### ParB2/ParS2 labels both *Ubx* ROIs with superior efficacy but with sensitivity to design

Partitioning (Par) proteins are essential components of a partitioning mechanism that ensures efficient division of bacterial chromosomes and plasmids to daughter cells (Khare et al., 2004). One of these Par proteins (ParB) is a DNA-binding protein with a centromere-like, sequence-specific binding site (ParS), which, like LacI/LacO, can be utilized as an FP labeling system. Following a strategy developed by Saad et al. to label genomic loci in yeast, we utilized ParB and ParS sequences from *Burkholderia cenocepacia* chromosomes 2 (c2) and 3 (c3) (Saad et al., 2014; Chen et al., 2017; Germier et al., 2018). The independent chromosomes are partitioned by a unique ParB protein, which recognizes the chromosome-specific ParS binding site. Unlike LacI, ParB spot formation depends on protein-protein interactions to recruit ParB molecules beyond those that bind directly to the ParS binding sites (Figure 3A, right). Following the terminology used by Saad et al., we refer to these complementary systems as ParB1/ParS1 (for c2) and ParB2/ParS2 (for c3). An ∼1 kb genomic sequence from each chromosome containing either four ParS1 or ParS2 sequences with intervening sequence—also termed INT1/ANCH1 and INT2/ANCH2, respectively—was used as the inserted DNA tag (Figures 3A and 4A) (Saad et al., 2014; Germier et al., 2018; Chen et al., 2018b). Successful genome labeling with ParB/ParS in *Drosophila melanogaster* using a truncated ParS2 containing three binding sites and coupled with a ParB2-GFP has been demonstrated previously in embryos (Chen et al., 2018b;). Here we demonstrate its use in imaginal discs and, as with LacI/LacO, demonstrate its utility in varying cellular and genomic contexts. We further characterize the orthogonal ParB system, ParB1, whose utility has yet to be demonstrated in *Drosophila* (Figure 4).

As with LacI, ParB2 was tagged C-terminally with a HaloTag and linker L1, or a Neon FP fusion and linker L1 or L2, allowing comparisons of efficacy between FP and linker (Figure 3A). In contrast to LacI, greater variability existed between constructs, suggesting a greater sensitivity to construct design: labeling efficiency varied 9-fold (from a mean of 0.03–0.28) and SNR varied 10-fold (from a mean of 5.4–53.8) (Figure S4). In fact, although spots can be detected in all contexts using ParB2-HaloTag (L1), its efficiency and SNR are poor enough to functionally prohibit its regular use. In particular, ParB2-HaloTag (L1) appears highly sensitive to genomic context as both metrics are reduced in the wing compared with the haltere at both ROIs (Figure S4). The relative improvement in efficacy with ParB2-Neon (L1) fusions suggests that at least part of the failure is intrinsic to the HaloTag. However, we cannot rule out the contribution of the linker, as further improvements are observed between ParB2-Neon (L1) and ParB2-Neon (L2) (Figures 4E and S4). Increased sensitivity to FP design of ParB2 as compared with LacI is likely due to the fact ParB spot formation requires both protein-DNA and protein-protein interactions, whereas LacI spot formation only requires protein-DNA interaction. Linker and FP choice can have profound effects on what are already weak protein-protein interactions between ParB molecules.

Despite the variability between ParB2 FP constructs, ParB2-Neon (L2), which overall displayed the highest efficiency and SNR, could effectively label all ROIs tested (Figure 3B). At ROIs in accessible (*abx* in haltere) and inaccessible (*abx* in wing) regions, ParB2-Neon (L2) exhibited high efficiency and SNR, though with a slight but statistically significant decrease in SNR in wing cells (Figures 3C and 3D). We detect this decrease in the wing at both the inaccessible *abx* and the accessible *UbxP*, suggesting that ParB2 foci formation is sensitive to unknown factors beyond accessibility. Furthermore, both ROIs within a transcribed body (*abx* in haltere) and outside (*UbxP* in haltere) were labeled with similar efficiency and SNR.

A direct comparison of LacI and ParB2 FP systems reveals that the best construct in each system (LacI-HaloTag (L1) and ParB2-Neon (L2)) exhibit comparable labeling efficiencies (Figures 2F and 3D), but ParB2 forms spots with a much higher SNR (Figures 2E and 3C). ParB2, unlike LacI, induces nucleation at the ROI through protein-protein interactions, which likely results in the recruitment of more FP molecules to the ROI and a brighter spot. However, despite the superior SNR of ParB2-Neon (L2), overall ParB2 FPs are more sensitive to fusion design compared with LacI, which are exacerbated by the genomic context of the targeted ROI. Thus, in contrast to LacI, for which we have a system that is detectable in three channels, our results have only identified a single workable ParB2 FP (Neon). Further optimization of the HaloTag linker and/or tests of additional FPs are necessary to expand this toolkit.

### Labeling genomic loci with a third system, ParB1/ParS1

The success of LacI and ParB2 FP labeling allows for a two-color labeling system that can be used to simultaneously image two tagged ROIs. This can be used, as we demonstrate below, to locate two genomic positions relative to one another; however, we sought to test a third FP to allow users the potential to image three genomic positions in parallel. Toward this end, we optimized the use of the orthogonal ParB system, ParB1/ParS1, which has yet to be used for labeling in *Drosophila* cells. Attempts at using ParB1 fusions (ParB1-Cherry, no NLS) that were successful in yeast failed in *Drosophila*, leading us to perform a similar optimization of both Linker and FP as we did for LacI and ParB2 (Saad et al., 2014). As before, ParB1 was tagged C-terminally with either a HaloTag utilizing linker L1 or a Neon FP fusion utilizing linkers L1 and L2, allowing comparisons of efficacy between FP and linker (Figure 4A). Interestingly, ParB1 shares features with both LacI and ParB2. Similar to ParB2, ParB1 exhibited great variability between constructs and ROI, particularly in terms of efficiency. Labeling efficiency varied from a mean of 0.04 to 0.42 (12-fold change), and SNR varied much less from a mean of 11.1 to 21.1 (1.9-fold change) (Figures 4B–4D and S5). The difference in variability between efficiency and SNR suggests that even in contexts where ParB1 labels inefficiently, when foci form they are nearly as bright as in contexts where ParB1 labels more efficiently.

Based on our results, the variability in ParB1 efficiency is primarily a product of FP choice and tissue context. Most notably, an often severe drop in efficiency was observed when comparing haltere and wing cells, interestingly both at the intronic enhancer, *abx*, and *UbxP*. While chromatin accessibility is vastly different between these tissues at *abx*, it is not so at *UbxP*, suggesting that a transcriptional OFF state even in the presence of chromatin accessibility affects ParB1 foci formation. Similar to our observations with ParB2, factors beyond accessibility can affect ParB1 foci formation. Finally, ParB1 behaved more like LacI than ParB2 in terms of sensitivity to linker. Neither the efficiency nor SNR of ParB1 was significantly altered when comparing L1 and L2 (Figure 4E).

In the process of testing our ParB1-FP fusions, we observed the formation of nuclear aggregates when ParB1 was fused to Neon but not HaloTag (Figure S5A). The formation of aggregates did not always prevent foci formation because nuclei were observed with both aggregation as well as a distinct focus, but perhaps it contributed to the average decrease in efficiency between ParB1-HaloTag constructs and ParB1-Neon constructs. Data presented in Figure 4 (excluding Figure 4E) utilize the ParB1-HaloTag construct for these reasons. Although our experimentation with FPs has not gone beyond the use of HaloTag and Neon, it should not be challenging to replace one of these FPs with a red-fluorescing protein, particularly in LacI constructs, which are least sensitive to FP and linkers to allow for three FPs each labeled with a distinct color.

During our analysis we observed nuclei with two spots. While this occurred for LacI, ParB2, and ParB1, it was much more frequent when using ParB1 and ParB2 compared with LacI. Because all experiments were conducted in the presence of only a single-tagged allele, the presence of two spots was likely the result of labeled sister chromatids. To address this hypothesis, we turned to the established Fly-FUCCI system that uses fluorophore-tagged degrons from the Cyclin B and E2F1 proteins to fluorescently distinguish cells in the G_1_ (green), S (red), and G_2_ (green and red) phases of interphase (Zielke et al., 2014) (Figure S6A). Because we combined Fly-FUCCI with our ParB2-Neon FP labeling system, which also fluoresces in the green channel, we restricted our analysis to the absence (G_1_) or presence (S and G_2_) of red fluorescence to represent the likely genome copy number: 1× and 2×, respectively. All observed nuclei with two ParB2-Neon spots also exhibited red fluorescence, supporting our initial hypothesis. However, it should be noted that not all red-fluorescing cells produce two spots. These cells are either in the process of synthesis and have not yet replicated the tagged ROI, or the efficiency of labeling is not sufficient to consistently label both sister chromatids (Figure S6B).

### LacI, but not ParB1 or ParB2, FP labeling can perturb transcription

**Figure 5:**
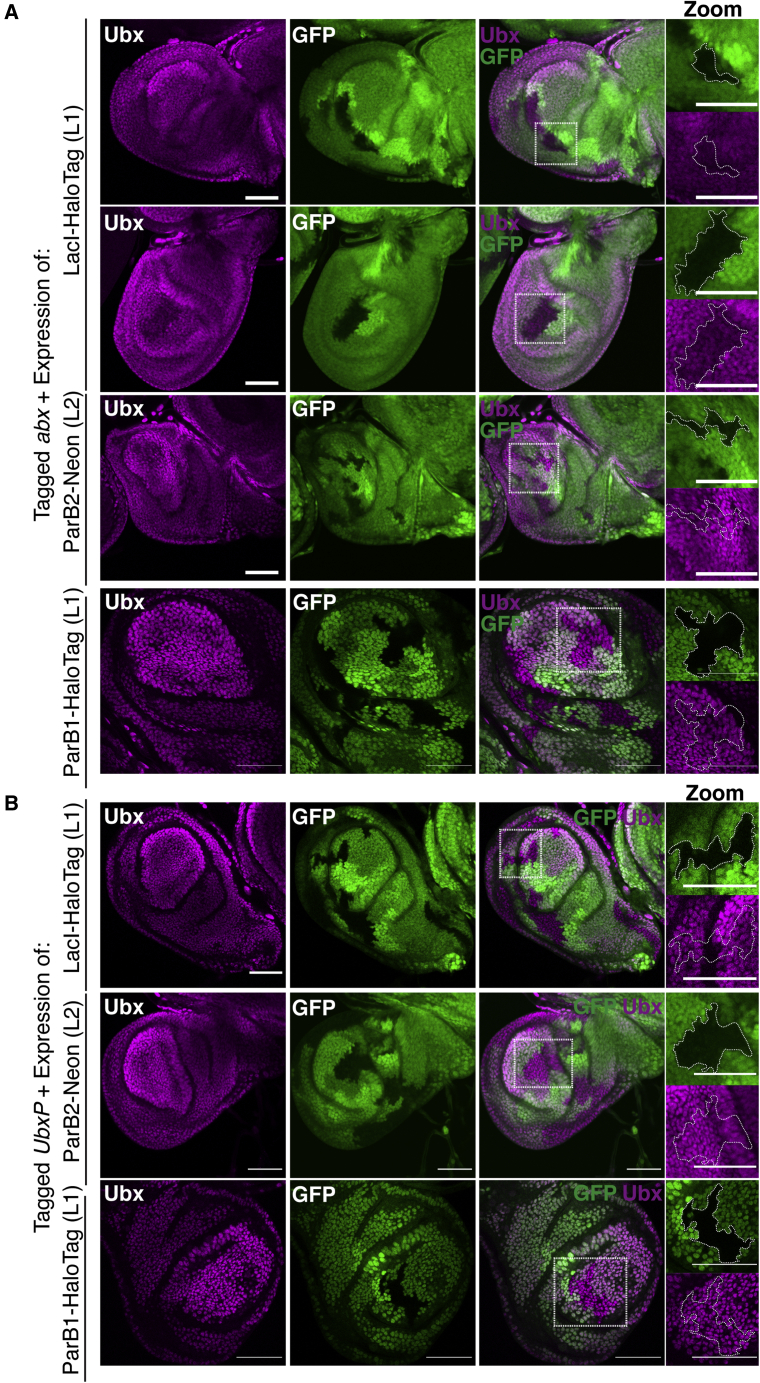
Clonal analysis of the effects of FP labeling on *Ubx* expression (A) Mitotic clonal analysis of FP labeling at *abx*. Top two panels: clones homozygous for 20× LacO *abx* with one copy of LacI-HaloTag (L1). Third panel: clones homozygous for ParS2-*abx* with one copy of ParB2-Neon. Fourth panel: clones homozygous for ParS1-*abx* with one copy of ParB1-HaloTag (L1). Quantification of clones with reduced Ubx: 4/8 LIHT2 clones, 0/13 B2N2 clones, 0/11 B1HT1 clones. (B) Mitotic clonal analysis of FP labeling at *Ubx* promoter. Top panel: clones homozygous for 20× LacO *UbxP* with one copy of LacI-HaloTag (L1). Middle panel: clones homozygous for ParS2-*UbxP* with one copy of ParB2-Neon (L2). Bottom panel: clones homozygous for ParS1-*UbxP* with one copy of ParB1-HaloTag (L1). Quantification of clones with reduced Ubx: 0/16 LIHT2 clones, 0/12 B2N2 clones, 0/2 B1HT1 clones. All clones were made 48 h after egg laying. Ubx immunostain, native GFP fluorescence, and a MERGE are shown, with zoom of a single clone. Scale bars, 50 μm.

Detection of genomic loci with FP labeling systems relies on the insertion of an exogenous DNA tag at an ROI and the expression and targeting of a heterologous protein to that ROI. Thus, they carry the risk of perturbing gene expression. This is particularly true when targeting an ROI within a transcribed gene body, as we are doing for *abx*. We tested the impact of FP labeling on *Ubx* expression. We generated mitotic clones homozygous for a *Ubx* allele tagged with 20× LacO, ParS2, or ParS1 at either *abx* or *UbxP* (Figure 5). LacI-HaloTag (L1), ParB2-Neon (L2), and ParB1-HaloTag (L1) were expressed, respectively. As expected, labeling of *UbxP* upstream of the gene body with all FP systems had no effect on *Ubx* expression in haltere discs (Figure 5B). Furthermore, *Ubx* expression was unaffected when the intronic *abx* was labeled with ParB2/ParS2 and ParB1/ParS1 (Figure 5A, bottom two panels). In contrast, a reduction in Ubx protein was observed in some, but not all, clones with homozygous LacI/LacO labeling (Figure 5A, top and middle panels). Importantly, insertion of the LacO array alone (with no LacI expression) did not affect *Ubx* expression, suggesting that modification of the DNA did not perturb necessary gene regulatory information (Figure S6C). Instead, it is likely that bound LacI interrupts transcription elongation. This is further supported by single-molecule FISH (smFISH), which assays nascent RNA production with a probe against the first intron of *Ubx*. In contrast to the decrease in Ubx protein levels in clones, no obvious reduction of FISH signal was observed, further providing evidence that transcription initiation is intact while elongation through the LacI-bound *abx* is affected, thereby reducing total protein levels (Figure S6D).

In contrast, the lack of perturbation of *Ubx* gene expression when labeling with both ParB systems suggests that spot formation can occur with a weaker protein-DNA interaction that does not impede the elongation of RNAPII. This is consistent with results from ParB labeling in yeast, in which it was found that transcription dominates over ParB binding and that the insertion of ParS sequences does not perturb normal nucleosome functioning, as evidenced by local H3 deposition levels (Saad et al., 2014). Thus, the nucleation of ParB at the ROI through protein-protein interactions benefits spot detection through increasing SNR without negatively affecting gene expression in the surrounding area.

### Enhancer-promoter distance at the *Ubx* Locus is decoupled from gene expression level

**Figure 6:**
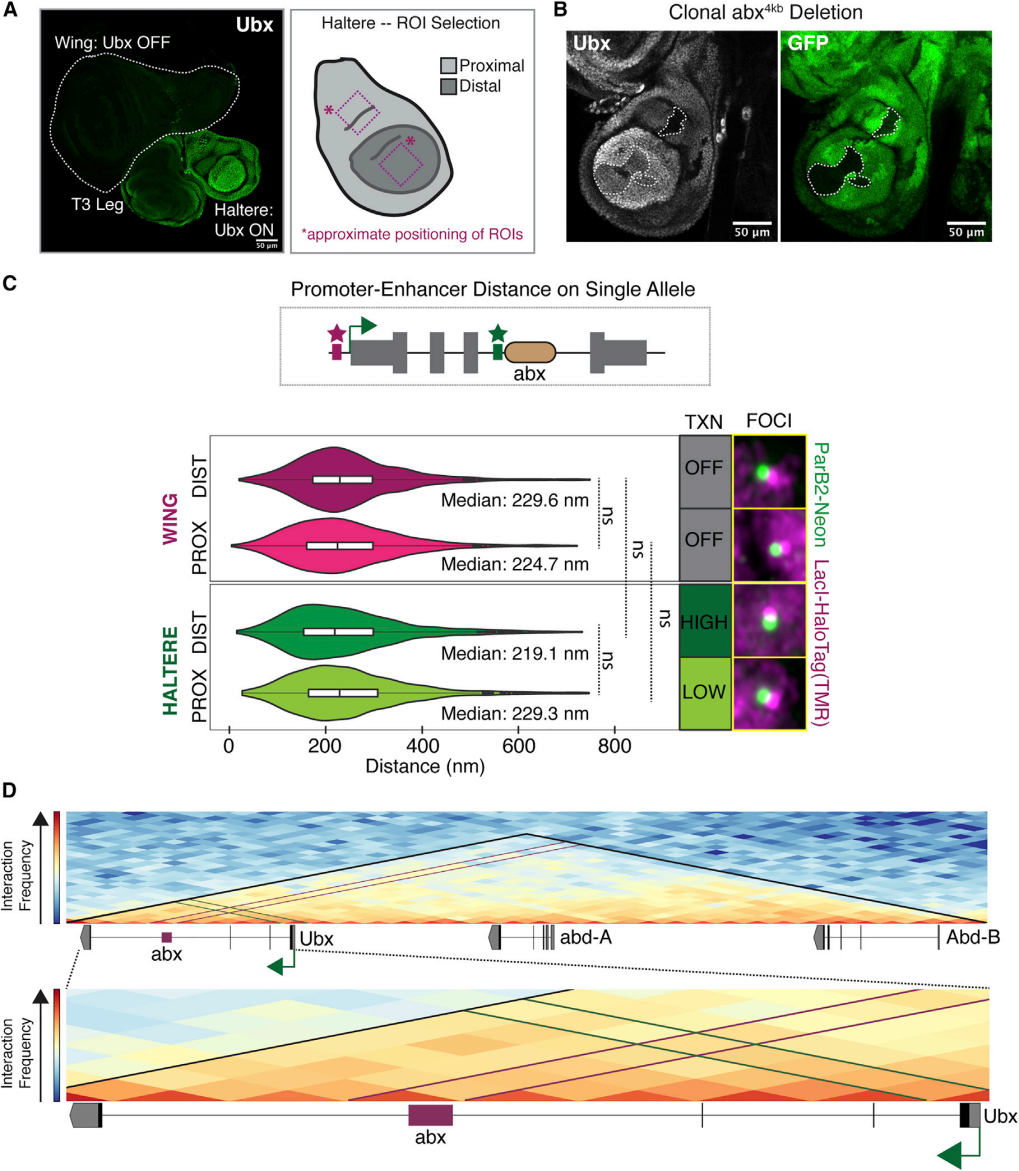
Assaying enhancer-promoter distance within the *Ubx* locus (A) (Left) Ubx immunostain in imaginal discs. A wing (white dotted line), T3 leg, and haltere disc are shown. (Right) Schematic of haltere disc with high distal Ubx and low proximal Ubx. Dotted boxes show approximate position of proximal, distal ROIs. Scale bar, 50 μm. (B) Ubx immunostain in haltere discs with 4 kb deletion of *abx* encompassing major FAIRE peaks. GFP marks clones, outlined with dotted line. Quantification of clones shows 14/32 clones with reduced Ubx and 5/32 with no Ubx. All other clones show normal Ubx. Scale bars, 50 μm. (C) Distribution of distances between *UbxP* and *abx*. A schematic shows labeling of single Ubx allele with LacI-HaloTag (L1) (magenta) and ParB2-Neon (L2) (green). Median values and *Ubx* transcription level are stated. Foci images shown are representative pairs with a measured distance approximating the median. Statistical significance was tested using a one-way ANOVA followed by Tukey’s multiple comparisons test with *α* = 0.05 in GraphPad Prism. ns, not significant. (D) Normalized observed/expected Hi-C interaction frequency matrix of the bithorax complex (top) and *Ubx* locus (bottom) in wing discs at 10 kb resolution. The interaction frequency represents the number of contacts between genomic loci captured with Hi-C. The TAD encompassing the bithorax complex is denoted by black lines. The approximate position of *abx* (red box) is defined by ATAC-seq peaks (Loker et al., 2021). The *Ubx* promoter is shown by the green arrow next to the first exon.

*Ubx*, required during both embryonic and larval stages of *D*. *melanogaster*, is well known for its role in specifying the identity of the haltere-bearing third thoracic segment (T3) instead of the wing-bearing second thoracic segment (T2). Wing development requires that *Ubx* be maintained in an OFF state, while haltere development requires that *Ubx* be maintained in an ON state (Simon et al., 1990; Pavlopoulos and Akam, 2011; Delker et al., 2019; Weatherbee et al., 1998; White and Akam, 1985; Bender et al., 1983; White and Wilcox, 1984, 1985; Lewis, 1978). In addition, the levels of *Ubx* expression vary within the ON state of the haltere imaginal disc: cells fated to develop into the distal appendage exhibit high *Ubx* expression, whereas proximal fated cells exhibit low *Ubx* expression (Delker et al., 2019) (Figure 6A). Together, these three expression states provide a platform to interrogate whether enhancer-promoter distances vary with expression level.

We used two of the FP systems—LacI-HaloTag (L1) and ParB2-Neon (L2)—to interrogate the relationship between enhancer-promoter distance within the *Ubx* locus and gene expression level. While a Ubx OFF state is mediated by the evolutionarily conserved Polycomb group of proteins, the variable Ubx ON states (HIGH versus LOW) are mediated by an autoregulatory transcriptional feedback loop that downregulates *Ubx* expression ∼1.7-fold specifically in the proximal haltere relative to the distal haltere (Delker et al., 2019; Coleman and Struhl, 2017; Beuchle et al., 2001). Reduction of Ubx protein, or loss of its proximally expressed cofactors, Hth/Exd, leads to upregulation of *Ubx* expression to distal levels, and enhancer-reporter transgene analysis revealed that the activity of this transcription factor complex can be in part explained by its binding to the intronic CRM, *abx*, ∼45 kb away from the promoter (Delker et al., 2019). While these data establish the existence of repressive elements within *abx* that are utilized in the proximal haltere, *abx* is also essential for establishing proper Ubx levels both distally and proximally. Clonal deletion of a ∼4 kb region of *abx* encompassing the major chromatin-accessible regions (Figure 1B) results in a decrease in Ubx levels ranging from slight reductions to complete loss, both proximally and distally (Figure 6B) (Delker et al., 2019). In addition, reporter transgenes containing fragments of *abx* coupled with a minimal promoter drive reporter expression in a pattern that recapitulates *Ubx* expression, including differences in level (Delker et al., 2019). Deletion of *abx* could remove elements directly necessary for activator binding, in addition to elements that indirectly affect transcription (e.g., by altering chromatin structure). Because *abx-*reporter transgenes recapitulate *Ubx* expression, we favor a model in which Ubx-mediated downregulation of *Ubx* transcription occurs by tuning down an inherent activating potential of *abx*.

If this model were true, one potential mechanism that could explain this “anti-activation” phenomenon is the regulation of enhancer-promoter distance. Distal transcriptional regulatory elements are able to modulate transcription levels at their associated promoter despite often large intervening distance. Looping of the chromatin to bring the CRM into proximity with the promoter can overcome this barrier by collapsing this distance in 3D space (Bonev and Cavalli, 2016). Forced looping studies, as well as live imaging of enhancer-promoter distance and transcriptional bursting, have further supported the causal role played by looping in transcriptional activation, and work on the *Drosophila* repressor, Snail, proposed the inhibition of looping (“anti-looping”) as a mechanism for transcriptional repression (Bartman et al., 2016; Chen et al., 2018b; Agelopoulos et al., 2012; Chopra et al., 2012).

We hypothesized that negative autoregulation of *Ubx* expression in the proximal haltere could be explained by TF-mediated altered distance between *abx* and the *Ubx* promoter, potentially decreasing the interaction between these two elements. To address this idea, we dual-labeled a single *Ubx* allele, knocking in 20× LacO at *UbxP* and ParS2 at *abx* using a combination of PhiC31- and BxB1-mediated RMCE events (Figures 1 and 6C). Co-expression of LacI-HaloTag (L1) and ParB2-Neon (L2) enabled us to locate the position of each ROI within the nuclei of a population of distal and proximal haltere imaginal disc cells, and subsequently measure the intervening 3D distance. Unexpectedly, by analyzing ∼1,000 (range 934–1,422) nuclei per cell population, aggregated from several independent imaginal discs, we found no significant difference in the 3D distance between *UbxP* and *abx* when comparing Ubx HIGH expressing distal cells and Ubx LOW expressing proximal cells (Figure 6C, bottom). Furthermore, a comparison of haltere (Ubx ON) and wing (Ubx OFF) cells again showed no significant difference (Figure 6C). Notably, although averaged over many cells and time, these distances likely represent true interactions because the entire *Ubx* locus, including *abx* and *UbxP*, is within a topological associated domain (TAD) identified by HiC in wing discs, which is a complementary method that reveals long-distance interactions between genomic sequences (Figure 6D) (Kribelbauer et al., 2020). Together, these data suggest that for *Ubx*, enhancer-promoter distance is decoupled from gene expression level.

### Inter-allelic distance between *Ubx* alleles correlates with expression level

**Figure 7:**
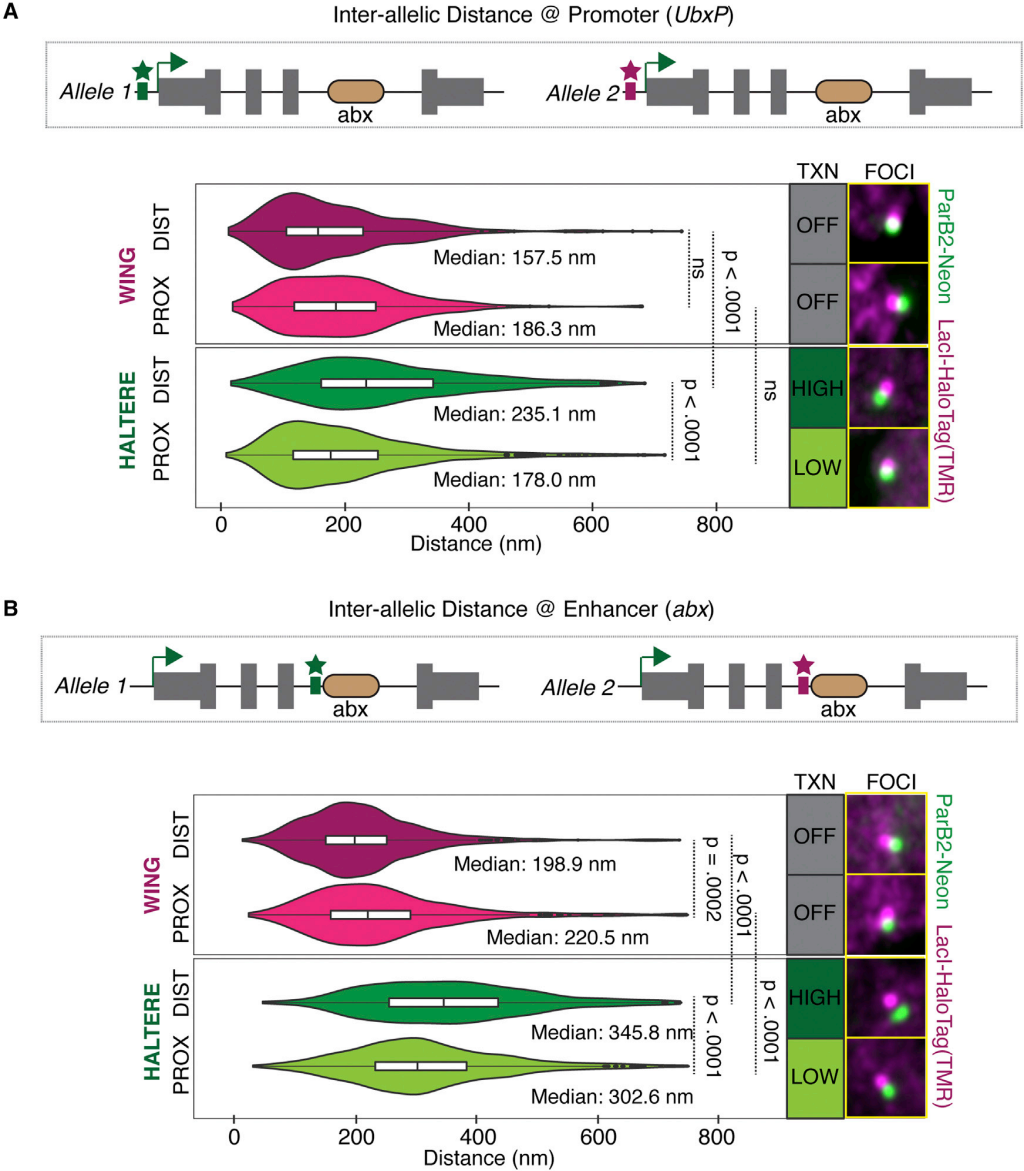
Assaying inter-allelic distance at the *Ubx* locus (A) Distribution of inter-allelic distances between *Ubx* alleles, measured at *UbxP*. A schematic shows labeling of each allele with LacI-HaloTag (L1) (magenta) and ParB2-Neon (L2) (green). (B) Distribution of inter-allelic distances between *Ubx* alleles, measured at *abx*. A schematic shows labeling of each allele with LacI-HaloTag (L1) (magenta) and ParB2-Neon (L2) (green). For both (A) and (B), median values and *Ubx* transcription state are stated. Foci images shown are representative pairs with a measured distance approximating the median. Statistical significance was tested using a one-way ANOVA followed by Tukey’s multiple comparisons test with *α* = 0.05 in GraphPad Prism.

While our studies of *UbxP-abx* distances utilized double targeting of a single *Ubx* allele, the tools we developed also enable us to study distances between alleles (“inter-allelic”) on homologous chromosomes, labeling each allele with a different FP system and, thus, a distinct color. We measured inter-allelic distance both as the distance between promoters (Figure 7A) and as the distance between *abx* enhancers (Figure 7B). Due to the somatic pairing of homologous chromosomes in *Drosophila*, a process termed transvection, regulation of promoters by CRMs can occur in *trans* in the absence of a CRM in *cis* (Kennison and Southworth, 2012; Mellert and Truman, 2012). Thus, closeness, proximity of *Ubx* alleles, was expected (Figure 7). However, unexpectedly and distinct from our measurements of enhancer-promoter distances within a single allele, we found that the distance between alleles is positively correlated with expression level: inter-allelic distance in distal haltere cells (*Ubx* HIGH) is larger than that observed in both distal wing cells (*Ubx* OFF) and proximal haltere cells (*Ubx* LOW) (Figures 7A and 7B). These differences hold true whether we measure the distance between homologous promoters (Figure 7A) or homologous *abx* CRMs, but are more pronounced at *abx* (Figure 7B). Similarly, an additional statistically significant increase in distance can be detected at *abx* when comparing proximal wing cells (*Ubx* OFF) and proximal haltere cells (*Ubx* LOW), which is not apparent at *UbxP*. We also note that differences in inter-allelic distance can be observed when comparing the distal wing and proximal wing, which both exhibit a *Ubx* OFF state, particularly at *abx* (Figure 7B).

## Discussion

We examined three DNA-binding FP fusion proteins—LacI, ParB1, and ParB2—to visualize the subnuclear position of genomic ROIs. We characterized the efficacy of each of system in *Drosophila* third instar imaginal discs. While we did not conduct live imaging, each system should be amenable to tracking ROIs in live tissue in addition to static imaging. For our studies of *Ubx* gene expression level, we used two orthogonal FPs—LacI-HaloTag (L1) and ParB2-Neon (L2)—and orthogonal GE techniques—PhiC31 and Bxb1—to label two genomic ROIs with distinct colors. With direct CRISPR insertion of DNA tags and the demonstrated efficacy of ParB1, it should be possible to simultaneously image three ROIs. We used HaloTag (HaloLigand TMR) and Neon fusions, but the availability of alternative fluorescent proteins and Halo ligands make it easy to expand the tools beyond the FPs used here.

Each FP system carries unique benefits and constraints that should be considered when designing imaging projects. The differences observed between systems likely stem from different mechanisms of spot formation. While LacI relies only on protein-DNA interactions, ParB FPs use both protein-DNA and protein-protein interactions to nucleate additional ParB2 FP molecules to the ROI, and thus may be affected more readily by construct design and chromatin context.

In general, labeling with LacI FPs is more robust to design and in ROI context. Labeling with ParB2 FPs holds the possibility of very high SNR and efficiency regardless of ROI context, but is sensitive to the design of the ParB2 FP. FP and linker used for construct design can have a large impact on SNR, labeling efficiency, and robustness in ROI context. ParB1, while resistant to changes in linker like LacI, also displays sensitivity to ROI context and FP used. The sensitivity of ParB1 and ParB2 to ROI context extended beyond chromatin accessibility. Both (although ParB1 to a larger extent) performed worse in the wing at both inaccessible (*abx*) and accessible (*UbxP*) regions. We speculate that this could be due to the presence of bound proteins despite local accessibility at the *UbxP*, or to the lack of nearby accessible regions that are needed for ParB nucleation.

This difference in foci-forming mechanism also likely contributes to the impact of FP binding on transcription. Although labeling ROIs outside of the *Ubx* gene body did not interfere with *Ubx* expression, binding of LacI FP, but not ParB1 or ParB2 FPs, at a transcribed ROI (*abx*) interfered with transcription. In ParB spot formation, protein-protein interactions are likely weak and protein-DNA interactions limited such that the ParB/DNA complex may be readily outcompeted by other cellular machinery, such as RNAP, and reform quickly. However, as the size of the region targeted increases, the accuracy of our estimation of the subnuclear location of the ROI decreases. Even though the incorporated ParS DNA tag is only slightly larger than the 20× LacO DNA tag, spreading of ParB beyond the DNA tag can result in increased efficacy of the labeling system as seen especially with ParB2, but with decreased accuracy.

Finally, we used two of these FPs—LacI-HaloTag (L1) and ParB2Neon (L2)—to interrogate the relationship between gene expression level and enhancer-promoter distance in *cis*, as well as inter-allelic distance at the *Ubx* locus. We first asked whether increased enhancer-promoter distance could account for the observed downregulation of *Ubx* expression by Ubx/Hth/Exd in proximal as compared with distal cells (Delker et al., 2019). Unexpectedly, we found that gene expression level and *UbxP*-*abx* distance are decoupled: a population of Ubx HIGH expressing distal haltere cells produces a similar distribution of enhancer-promoter distances as a population of Ubx LOW expressing proximal haltere cells. This relationship even holds true when comparing Ubx OFF wing cells with Ubx ON haltere cells. We cannot say if the observed distance is necessary for a Ubx ON state or whether it represents a true enhancer-promoter loop, only that it does not vary with expression level. This suggests that the mechanism of Ubx/Hth/Exd negative autoregulation in proximal cells does not depend on modulating the distance between *abx* and the promoter and is consistent with the observation that the entire locus is within a single TAD, at least in wing nuclei. Decoupling of enhancer-promoter distance and transcriptional activation was reported at the *Sox2* locus in murine embryonic stem cells using live imaging (Alexander et al., 2019); other reports find clear correlations between enhancer-promoter distance and transcription, raising the possibility that functional enhancer-promoter distances may differ between genes and tissues (Chen et al., 2013).

In contrast to enhancer-promoter distances, we found that inter-allelic distance increased in accordance with transcriptional activity. This was particularly notable when measuring distance at the intronic *abx* but also occurred to a lesser extent at the promoter. Future studies are required to determine whether inter-allelic distance of *Ubx* is a cause or consequence of transcription.

### Limitations of the study

In principle these tools can be used to label ROIs in live cells, although we have not tested them in this context. Doing so is important to determine whether the range of distances we measured in our experiments over a population of cells is representative of the range of distances that occur within a single cell over time. We also note that although some of these distance measurements correlate with the levels of transcription, our experiments do not address causality.

## STAR★Methods

### Key resources table

**Table:** 

REAGENT or RESOURCE	SOURCE	IDENTIFIER
**Antibodies**		

Mouse anti-Ubx	Developmental Studies Hybridoma Bank	Cat# FP3.3858; RRID:AB_10805300

**Bacterial and virus strains**		

XL1 Blue Cells	Agilent	Cat#200249
Stbl2 Cells	ThermoFisher	Cat#10268019

**Biological samples**		

*Drosophila melanogaster* 3rd Instar Larval Wing Discs	This Paper	N/A
*Drosophila melanogaster* 3rd Instar Larval Haltere Discs	This Paper	N/A

**Chemicals, peptides, and recombinant proteins**		

Halo Ligand-TMR	Promega	Cat#G8251

**Deposited data**		

Published FAIRE-Seq Data	McKay and Lieb (2013)	GEO: GSE38727
Published ATAC-Seq Data	Loker et al. (2021)	GEO: GSE166714
Published Hi-C Data	Kribelbauer et al. (2020)	GEO: GSM3927691

**Experimental models: Organisms/strains**		

*Drosophila melanogaster*: nanos-Cas9	Fly Stocks of National Institute of Genetics (NIG-FLY)	CAS-0001
*Drosophila melanogaster*: yw122 (P{hsFLP}12)	Generated by Gary Struhl	N/A
*Drosophila melanogaster*: FRT82B (P{neoFRT}82B)	Bloomington	Chr III of Bloomington Cat#86313
*Drosophila melanogaster*: FRT82B ubiGFP	Bloomington	Chr III of Bloomington Cat#5188
*Drosophila melanogaster*: w[1118]; Kr[If-1]/CyO, P{ry[+t7.2]=en1}wg[en11]; P{w[+mC]=Ubi-GFP.E2f1.1-230}5 P{w[+mC]=Ubi-mRFP1.NLS.CycB.1-266}12/TM6B, Tb[1].	Bloomington	Cat#55124
*Drosophila melanogaster*: 256X LacO	Bloomington	Cat#25371
*Drosophila melanogaster*: Novel Strains for this Study	This Study	Table S1

**Oligonucleotides**		

Novel Oligonucleotides and Sequences	This Study	Table S1
Ubx smRNA FISH Probes	Purchased from Biosearch Technologies	Table S2
Additional Sequences for Constructs Used	This Study	Table S3

**Recombinant DNA**		

pCFD4	Addgene	Cat#49411
pFG2 (ParS1)	Addgene	Cat#87250
pFG4 (ParS2)	Addgene	Cat#87251
pRVV54 (tdtomato enhancer reporter)	Gift From Roumen Voutev	N/A
Plasmids for this Study	Our Lab	Table S1

**Software and algorithms**		

Signal to Noise Calculations: Fiji Macro and R Script	This Study	Zenodo: https://doi.org/10.5281/zenodo.5601964
3D Distance Calculations: FIJI Macros and R Script	This Study	Zenodo: https://doi.org/10.5281/zenodo.5601964
FIJI Image Processing	Schindelin et al. (2012)	N/A
Labeling Efficiency Calculations: Fiji Macro	This Study	Zenodo: https://doi.org/10.5281/zenodo.5601964

### Resource availability

#### Lead contact

Further information and requests for resources and reagents should be directed to and will be fulfilled by the lead contact, Richard Mann (rsm10@columbia.edu).

#### Materials availability

All plasmids and fly lines generated by this study will be shared by the lead contact upon request.

### Experimental model and subject details

The experimental model for this study is *Drosophila melanogaster*. A full list of strains used in this paper is included in the key resources table and Table S1. Flies were maintained at 25C on cornmeal food using standard laboratory techniques. The tissues analyzed throughout the paper consisted of wing imaginal discs and haltere imaginal discs dissected from 3^rd^ instar wandering larvae.

### Method details

#### CRISPR/Cas9 targeting of *abx* and *Ubx* Promoter/Exon1

Two regions within *Ubx* (the intronic CRM, *abx*, *Ubx* Promoter/Exon1) were targeted with CRISPR/Cas9. For each targeting event, two gRNAs were designed flanking the region of interest. gRNA sequences for each of the regions are as follows – *abx*: GAGATGCTTTTGAATTCTCG and GGCAGATCGGATTGGATCTT; *Ubx Promoter/Exon1*: GAATTCGAAGAAAATTAG and GTAAGACATATGAAAGC. gRNAs were cloned into the pCFD4 dual gRNA vector (http://www.crisprflydesign.org/, Port et al., 2014 (Port et al., 2014)). Homemade PhiC31 donor vectors were made containing either a ubiDsRED or P3-RFP fluorescent selection marker flanked by inverted PhiC31 attP recognition sequences – the ubiDsRED cassette contains a full attP sequence (GTACTGACGGACACACCGAAGCCCCGGCGGCAACCCTCAGCGGATGCCCCGGGGCTTCACGTTTTCCCAGGTCAGAAGCGGTTTTCGGGAGTAGTGCCCCAACTGGGGTAACCTTTGAGTTCTCTCAGTTGGGGGCGTAGGGTCGCCGACATGACACAAGGGGTTGTGACCGGGGTGGACACGTACGCGGGTGCTTACGACCGTCAGTCGCGCGAGCGCGA), whereas the P3-RFP cassette contains a minimal attP sequence (CCCCAACTGGGGTAACCTTTGAGTTCTCTCAGTTGGGGG) from Voutev et al. 2018 (Voutev and Mann, 2018). A homemade BxB1 donor vector was made with a ubiGFP fluorescent selection marker flanked by inverted BxB1 attP recognition sequences (GGTTTGTCTGGTCAACCACCGCGGTCTCAGTGGTGTACGGTACAAACC) from (Voutev and Mann, 2017). ∼1.5 kb homology arms were cloned on either side of the inverted attP sites. Primers used to clone the homology arms are as follows: *abx*: (Left Arm) GCCAGAAGCTGCAAATTCAAG and CTTTGGGTTCTGTTCCACAGC, (Right Arm) GAATTCAAAAGCATCTCCGCATAAAG and GCCAACCGCAGACTGTGCGA; *Ubx Promoter/Exon1*: (Left Arm) GCTCAACTGTAGTTTTCTGTTCG and ATTTTCTTCGAATTCTTATATGCTAT, (Right Arm) AGCAGGCAGAACAGACCTT and CTCGCAGAGATTGTCTGACAC. The gRNA template (pCFD4) and donor template were injected into a germline-expressing Cas9 strain (nanos-Cas9, Kondo et al. 2013 (Kondo and Ueda, 2013)) at a concentration of 250 ng/μL and 500 ng/μL, respectively. Selection of positive CRISPR events was done by screening for the presence of ubiDsRED, P3-RFP, or ubiGFP. Positive fly lines were validated by PCR and Southern Blot analysis. Additional details for the CRISPR/Cas9 protocol used can be found in Figures 1 and S1, and Table S1.

#### Recombinase mediated cassette exchange (RMCE)

PhiC31-mediated RMCE was used to replace the ubiDsRED/P3-RFP selection markers inserted using CRISPR/Cas9 into *abx and UbxP/Ubx Exon1*. BxB1-mediated RMCE was used to replace the ubiGFP selection markers inserted using CRISPR/Cas9 into *abx* when double targeting of the *Ubx* allele was desired. Specifically, we have used PhiC31 to replace both *abx* and *Ubx Promoter/Exon1* with all DNA tags (ParS1, ParS2, 20X LacO). We have used Bxb1 to replace *abx* with the ParS2 tag only, but there is no reason to expect it would not work for all other tags. For PhiC31 replacements, a homemade vector was used, containing inverted PhiC31 attB recognition sequences (CGGGTGCCAGGGCGTGCCCTTGGGCTCCCCGGGCGCGTAC) flanking a multiple cloning site for insertion of sequences used for replacement alleles. For Bxb1 replacements, a homemade vector was used, containing inverted Bxb1 attB recognition sequences (GGCTTGTCGACGACGGCGGTCTCCGTCGTCAGGATCAT) flanking a multiple cloning site for insertion of sequences used for replacement alleles (Voutev and Mann, 2017). Replacement with wildtype sequence from *abx* and *Ubx Promoter/Exon1* was performed by amplifying regions of interest from the yw genome. The following primers were used – *abx*: ATCCAATCCGATCTGCCCAG and TCGAGGAGTGAGTAAGAGATTGATAAAG; *Ubx Promoter/Exon 1*: TAGAGGTTGTATTGTTTTATTAATAAAAAACCTATTG and TTCATATGTCTTACATTACAAGTTGTTATCTGTTTTTCC. Replacement cassettes with wildtype *abx* plus DNA tag (ParS1/2, 20XLacO) were constructed by cloning the DNA tag into cloning sites downstream of the cloned *abx* wildtype sequence. Replacement cassettes with wildtype *Ubx Promoter/Exon1* plus DNA tag (ParS1/2, 20XLacO) were cloned through dividing genomic sequence into two fragments. Fragment 1 was amplified with the following primers: TAGAGGTTGTATTGTTTTATTAATAAAAAACCTATTG and GCTTACGCAAATTATTTGTATCTAATTC. Fragment 2 was amplified with the following primers: CATATTCTAGCACAAAGATTGGG and TTCATATGTCTTACATTACAAGTTGTTATC. The DNA tag was inserted into cloning sites between these two fragments such that the final constructed allele contains the insertion of the tag at ∼400nt upstream of the transcription start site of *Ubx*. The necessary recombinase enzymes, PhiC31 and Bxb1, were either injected as plasmid along with the donor cassette (PhiC31 *abx* replacements) or were expressed from a genomic insertion (nanos-PhiC31 on the X chromosome for *Ubx Promoter/Exon1* replacements and vasa-Bxb1(3’ nos) on the II chromosome for Bxb1 *abx* replacements (Voutev and Mann, 2017). Progeny from injected flies were screened for the loss of the fluorescent selection marker (ubiDsRED, P3-RFP, ubiGFP). Because the attP/attB reaction does not provide directionality, replacements can be inserted in the forward or reverse direction. Southern blot was performed to ensure the correct directionality of the replacement. Additional details for the RMCE protocol used are found in Figures 1 and S1, and Table S1.

#### DNA tag cloning

Construction of LacO repeats was conducted by taking advantage of the compatibility of PstI (CTGCAG) and NsiI (ATGCAT), along with an additional BamHI (GGATCC) cut site. An initial 36bp double stranded LacO sequence (CCACATGTGGAATTGTGAGCGGATAACAATTTGTGG) ((Robinett et al., 1996; Betz and Samsor, 2016; Nick et al., 1982) with a 5′ PstI half-site flank and a 3’ NsiI full site, BamHI half site flank was generated through synthesis and annealing of complementary forward and reverse single strand oligos. This double strand fragment was ligated into a vector with cut PstI and BamHI sites. Additional LacO units were inserted by ligating a PstI/BamHI cut LacO unit into an NsiI/BamHI cut vector. The BamHI site serves as an anchor to provide directionality, and the ligation of PstI and NsiI creates a hybrid site that is no longer able to be cut by either enzyme. TetO repeats were similarly constructed using a base TetO unit (TCCCTATCAGTGATAGAGA), but varying the spacer sequence in between (Spacer1: TCGGGCGATT, Spacer2: CTATAAGATT, Spacer3: CCGCATTGCG, Spacer4: TGCTGTCGGC). All repeats were cloned using Stbl2 cells; all other non-repetitive cloning was done using XL1-Blue Cells. ParS1 and ParS2 DNA tags were cloned from plasmids available on addgene (ParS1: pFG2, plasmid #87250; ParS2: pFG4, plasmid #87251). Sequences were amplified and cloned into the replacement cassettes as described above. ParS1 primers used are: CCATTCGGACGATCGG and CAGATCTGGCGCGCC. ParS2 primers used are: CAAATCCGGGGCGCT and CCGGCGTCAACTTCTATCTACTC. Strains and plasmids made, along with important oligos and sequences can be found in Table S1. We have additionally used a fly line that contains an insertion of 256X LacO. This line is available from Bloomington (Cat#25371). Some studies presented in Figure S2 were conducted on transgenic flies with a minimal *abx* sequence driving tdtomato expression. Various LacO repeat numbers were cloned upstream of the *abx* sequence. For these studies we used a pRVV54_tdtomato vector, which was a gift from Roumen Voutev, and inserted our transgene into attP40 on Chr II.

#### Expression of fluorescent protein (FP) fusions

Linkers and nuclear localization sequences used are displayed within the main figures. Each FP fusion was cloned into a homemade hsp70 expressing vector (Tables S1 and S3) such that FP expression was heat-shock inducible. Expression of FP fusions was induced with heat shock at 37C for 15 minutes. The optimal length of rest period at 25C following heat shock was empirically determined (Figure S2) to be 4 hours (Figure 2).

#### Dissection and staining of 3^rd^ instar imaginal discs

Wandering third instar larvae were collected and dissected in PBS to invert the head region and expose attached imaginal discs to solution. HaloLigand staining occurred at this stage by incubating inverted heads with a 2.5 uM solution of HaloLigand-TMR (Promega) in PBS for 20 minutes rocking at RT. HaloLigand solution was then removed, the inverted heads washed 2X with PBS for 5 min at RT to remove excess ligand. For static imaging, inverted heads were fixed in Fix Solution (PBS/4% Paraformaldehyde/.1% TritonX/.1% Sodium Deoxycholate) for 25 minutes at RT. Fix solution was removed and replaced with Staining Solution (PBS/.3% TritonX/1% BSA). Inverted heads were washed 2X with Staining Solution for 20 minutes at RT. The addition of DAPI (1:1000) in Staining Solution was carried out at RT for an incubation period of at least 30 min. This was followed by two washes with Staining Solution, dissection of discs from the inverted heads in PBS and mounting of the discs in Vectashield. For experiments that include an immunostain, primary antibody was incubated in Staining Solution overnight at 4C. This was washed 4X in Staining Solution at RT, followed by incubation with secondary antibody and DAPI for 1.5 hours at RT. Native Neon signal was acquired.

#### *Ubx* smFISH

A probe library containing 48 20-nt Stellaris FISH probes (listed in Table S2) was designed to target the first 2 kb of Ubx Intron 1. Libraries were ordered from Biosearch Technologies and labelled with Quasar 670. Wandering third instar larvae were collected and dissected in PBS to invert heads and expose discs to solution. Inverted heads were washed in PBSM (PBS/5mM MgCl2) 1X at RT, followed by fixation in PBSM/4% PFA for 10 min at RT. Discs were permeabilized with PBS/.5% TritonX for 10 min at RT and washed once with PBSM for 10 min at RT. Inverted heads were washed 1X with Pre-Hyb (10% deionized formamide in 2X SSC) for 10 min at RT prior to hybridization. Hybridization was performed overnight in a thermoshaker at 37°C (∼600RPM) covered in foil. Hybridization buffer contains: 2X SSC, .2 mg/mL BSA, 50% Dextran Sulfate, 10% deionized formamide, 50 μg/mL *E*. *coli* tRNA, 50 μg/mL salmon sperm ssDNA, and 125 nM Ubx Intron Probe. 100 μL of hybridization buffer was used for each sample. The following day, hybridization buffer was removed and heads were washed with Pre-Hyb buffer for 20 minutes at 37°C and 20 min at RT. Inverted heads were washed with PBS for 10 min at RT, stained with DAPI (PBS/DAPI (1:1000 dilution) for 30 min at RT and resuspended in PBS. Discs were dissected from inverted heads in PBS/1%BSA and mounted in Vectashield prior to imaging.

#### Deletion of *abx*^*4kb*^

Experimental details can be found in detail in Delker, Ranade et al., 2019 (Delker et al., 2019). In brief, the gRNAs used for the replacement of abx4kb are: GGCTTTGCAACTAATTGAAA and GTAAATGTTGGCTATTCAAAA. Primers used for cloning the homology arms are: (Left Arm) GATGTAGGCCATGGTTTCGGC and TGAATAGCCAACATTTACTGACTCG, (Right Arm) AAACGGTAAAACTTGAGATTTTCTTATT and CGGAGAATCCGTATGAATCG. The ubi-dsRED PhiC31 cassette was used as described above. The *abx*^*4kb*^ deletion allele was generated by using an attB donor plasmid containing a multiple cloning site (gaagcttcctaggaggcctagatctgcggccgcttaattaaacgcgtgaatgggcgcgccgctagccatatgggtaccggatcc) to replace the ubi-dsRED cassette from CRISPR targeting. This MCS sequence serves as our deletion of the region.

#### Mitotic clones

Alleles of interest (wildtype replacements, tagged replacements, deletions) were recombined with standard FRT82B lines. Flies with mutant recombined alleles were crossed to FRT82B ubiGFP (to mark the clones) and progeny of this cross were heat shocked at 37C for 40min-1hr, 48 hours after egg laying (AEL). Wandering third instar larvae were collected 72 hours after heat shock, dissected, and subjected to immunostaining as described above. Native GFP fluorescence was acquired, and Ubx protein detected with an anti-Ubx antibody (mouse, 1:10 FP3.38 from Developmental Studies Hybridoma Bank (DSHB) in supernatant or ascites form. For clonal analysis of the effect of DNA tag and FP labeling on *Ubx* expression we expressed the FP fusion using the same protocol as before. A 15minute heat shock 4 hours prior to dissection was used. Because our FP fusion is heat shock inducible, it is possible that FP expression also occurred earlier during development during clone induction. However, this early expression did not impact *Ubx* expression later.

#### Microscopy setup

Imaging of discs was conducted on the following microscopes: Leica SP5 Confocal Microscope, and Zeiss LSM 800 Confocal Microscope with AiryScan. All DNA labeling images shown in the main figures were acquired on the Zeiss LSM 800 with AiryScan processing. All image visualization and processing was conducted in FIJI image processing software (Schindelin et al., 2012).

#### Published data accession

FAIRE, ATAC, and HI-C data shown was downloaded from NCBI GEO database with accession numbers listed above in data and code availability.

#### Labeling efficiency calculations

Images for each FP fusion at each ROI in each tissue were acquired on the Zeiss LSM 800 with Airyscan Processing following dissection and staining as stated above. A 63x/NA1.4 objective with an additional 2X zoom were used. LacI-HaloTag was stained with TMR prior to fixation and native Neon fluorescence was acquired. All images acquired were from the distal compartment of either the wing or haltere. Labeling efficiency was determined by analyzing a series of MAX projections of derived from subsections of a total Z-stack. Each Z-stack was divided into subsections of 10 slices (plus an additional subsection of the remaining slices if the total stack size is not divisible by 10). A MAX projection of each subsection was generated to generate a 2D representation of the 3D substack. For each MAX projection, nuclei were selected using the plugin “Cell Counter” in FIJI. Of the nuclei selected, those that were positive for FP spot formation were then counted. A ratio of spot+/total nuclei was determined. The results from each MAX projection represents a single data point in the analysis. Two to three Z-stacks were used for each condition. An in-house macro was written to streamline the division of Z-stacks into MAX projected subsections. Code has been deposited at Zenodo.

#### Signal-to-noise (SNR) calculations

Images for each FP fusion at each ROI in each tissue were acquired on the Zeiss LSM 800 with Airyscan Processing following dissection and staining as stated above. A 63x/NA1.4 objective with an additional 2X zoom were used. LacI-HaloTag was stained with TMR prior to fixation and native Neon fluorescence was acquired. All images acquired were from the distal compartment of either the wing or haltere. SNR calculations were performed using a formula from Chen et al., 2018 (Chen et al., 2018a) and automated using an in-house FIJI macro and R script (deposited at Zenodo). FP spots were manually selected throughout a Z-stack image. A 40x40 pixel square ROI is generated around each spot selected, and a 13 slice substack extracted. Within this substack, the z-slice with the max skew was identified and the MAX intensity and position of MAX intensity pixel identified in this slice using the “Measure” feature in FIJI. To obtain the average background signal, the spot was deleted within the max skew slice by deleting a 14x14 pixel box centered on the position of the pixel of MAX intensity (center of spot). A second 28x28 pixel box was generated with the same center, which was used to calculate the average signal and standard deviation. A minimum threshold was set to exclude the 0 values within the deleted 14x14 box. SNR is defined as (MAX intensity-Average Intensity)/Standard Deviation.

#### Distance calculations

3D distances were determined by identifying the center of each FP spot in 3D and calculating the 3D distance between pairs of spots. Green spots (ParB2-Neon(L2)) were picked within a Z-stack image. A 40x40 square ROI was generated around this selected spot and a 15-slice substack extracted for both the green channel and the red channel (LacI-HaloTag(L1) TMR). Exclusion of nuclei with two green or two red spots was done manually to avoid nuclei that have replicated their DNA. The X, Y, Z position of the green and red spot in each ROI was determined using the ‘3D Object Counter’ plugin in FIJI. Within 3D Object Counter, slice was set by identifying the slice with the highest max pixel intensity (representative of the slice with the center of the spot), threshold was set at .7∗max pixel intensity, and approximate volume was set at 20 pixels. An in-house FIJI macro was used to automate this process across all ROIs generated. For many of the datasets presented, position was also determined using the FIJI plugin, ‘TrackMate,’ using the DOG detector, an estimated spot diameter of 11 pixels, and sub-pixel localization. An in-house R script was used to calculate the 3D distance between the green and red spots given the X, Y, Z position of each. Distance was calculated as $\sqrt{{\left(x1-x2\right)}^{2}+{\left(y1-y2\right)}^{2}+{\left(z1-z2\right)}^{2}\;}\;$. Distance data presented in the paper is derived from the 3D Object Counter analysis, but distances calculated using Trackmate localization showed the same relative changes between tissue and/or compartment. Only distances below a threshold of 750 nm were kept for each dataset. This threshold was empirically determined to remove foci pairs that spanned neighboring nuclei rather than residing within the same nucleus. FIJI Macros and R Scripts have been deposited at Zenodo as reported in the key resources table.

#### Cell cycle analysis with fly FUCCI

FUCCI flies (Zielke et al., 2014) with the following genotype were obtained from Bloomington Fly Stocks (Stock #55124): *w*[*1118*]; *Kr*[*If-1*]*/CyO*, *P{ry*[+*t7*.*2*]*=en1}wg*[*en11*]; *P{w*[+*mC*]*=Ubi-GFP*.*E2f1*.*1-230}5 P{w*[+*mC*]*=Ubi-mRFP1*.*NLS*.*CycB*.*1-266}12/TM6B*, *Tb*[*1*]. These flies contain cassettes on chromosome III that drive ubiquitous expression of a nuclear localized GFP-E2f degron, and a nuclear localized mRFP-CycB degron. These flies were crossed with flies of the following genotype: *yw*; *hsp70-ParB2 Neon*(*L2*); *abx-ParS2*/Compound Balancer. Non-tubby 3^rd^ instar larvae were selected and dissected and stained as above. Because of the co-fluorescence of ParB2-Neon and GFP-E2F1, we restricted our analysis to the presence and absence of RFP signal. A schematic of this system is shown in Figure S6.

### Quantification and statistical analysis

For comparisons between two groups, data were analyzed by the Student’s t test. For comparisons among more than two groups, we utilized one-way analysis of variance (ANOVA) followed by Tukey’s multiple comparisons test. Reported p-values from the ANOVA analysis are adjusted p-values. All statistical analyses were performed using the GraphPad Prism software. The figure legend for each figure states exactly what tests were used for the data contained within that figure. Throughout the paper, adjusted p-values are either reported or the following symbols are used: ns = not significant, ∗ = p < .05, ∗∗ = p < .01, ∗∗∗ = p < .001, ∗∗∗∗ = p < .0001).

## Data Availability

• All microscopy data is available upon request by the lead contact. • All original code has been deposited at Zenodo and is publicly available as of the date of publication. DOIs are listed in the key resources table. • Any other information required to reanalyze the data reported in this paper is available from the lead contact upon request.

## Supplemental information

Document S1. Figures S1–S6

Table S1. Strains, plasmids, and sequences used in this study, related to key resources table

Table S2. Ubx single-molecule RNA FISH probes, related to key resources table

Table S3. Sequences of fluorescent proteins and tags used in this study, related to key resources table

Document S2. Article plus supplemental information
