# Supplementary material for: Fluorescent labeling of genomic loci in Drosophila imaginal discs with heterologous DNA-binding proteins
Source: Cell Rep Methods. 2022 Mar 9;2(3):100175. doi: 10.1016/j.crmeth.2022.100175 (PMC9017127; doi:10.1016/j.crmeth.2022.100175)
Supplement: Document S1. Figures S1–S6 [file mmc1.pdf]

Cell Reports Methods, Volume *2*

Supplemental information

Fluorescent labeling of genomic loci in *Drosophila* imaginal discs with heterologous DNA-binding proteins

Rebecca K. Delker, Ross H. Munce, Michelle Hu, and Richard S. Mann

Supplemental Figures

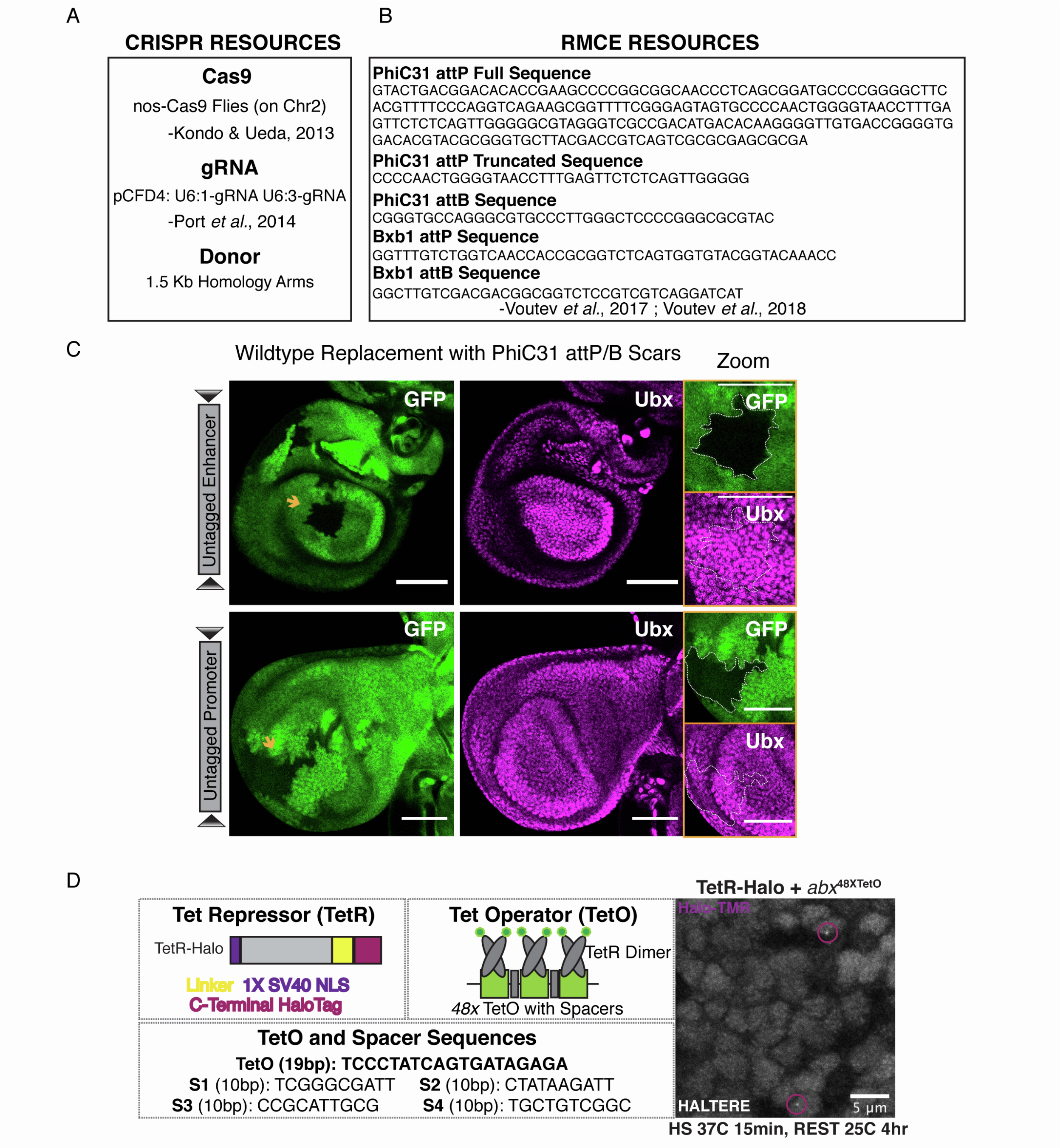

Supplemental Figure 1. Additional CRISPR/Cas9, RMCE Resources, and TetR/TetO Labeling of *abx*. Related to Figure 1.

(A) CRISPR resources and their references are given. nos-Cas9 flies used were first reported in Kondo *et al.* (Kondo and Ueda, 2013). The gRNA constructs used were developed by Port *et al.* (Port et al., 2014). And 1.5 kb homology arms were used for the donor cassette.
(B) Sequences for the recombinase attP and attB used. Each of these has been used before in the following references: Delker *et al*. (Delker et al., 2019), Voutev *et al*. 2018 (Voutev and Mann, 2018), and Voutev *et al.* 2017 (Voutev and Mann, 2017).
(C) Clonal analysis of untagged *abx* enhancer and *Ubx* promoter sequences to assess the impact of PhiC31 attP/attB scars on *Ubx* expression. Clones were made 48hr AEL. Native GFP fluorescence (green) is shown alongside a Ubx immunostain

(magenta). Zoomed images of single clones are shown in both the GFP and Ubx channels. Scale bars are 50 micron. A total of 8 *abx* clones and 11 *UbxP* clones were imaged. All clones displayed normal Ubx expression compared to surrounding tissue.
(D) A schematic of the Tet Repressor (TetR) FP fusion used and of its binding as a dimer to a 48x TetO DNA tag. Sequences of TetO and the spacers used between TetO units are shown. Expression of TetR-HaloTag with a 15min HS at 37C followed by a 4hr REST at 25C in the background of a 48x-TetO tagged *abx* allele. Labeling of TetO-*abx* with TetR-HaloTag using this protocol is very inefficient. Only two nuclei show spot formation (circled). Scale bar is 5 micron.

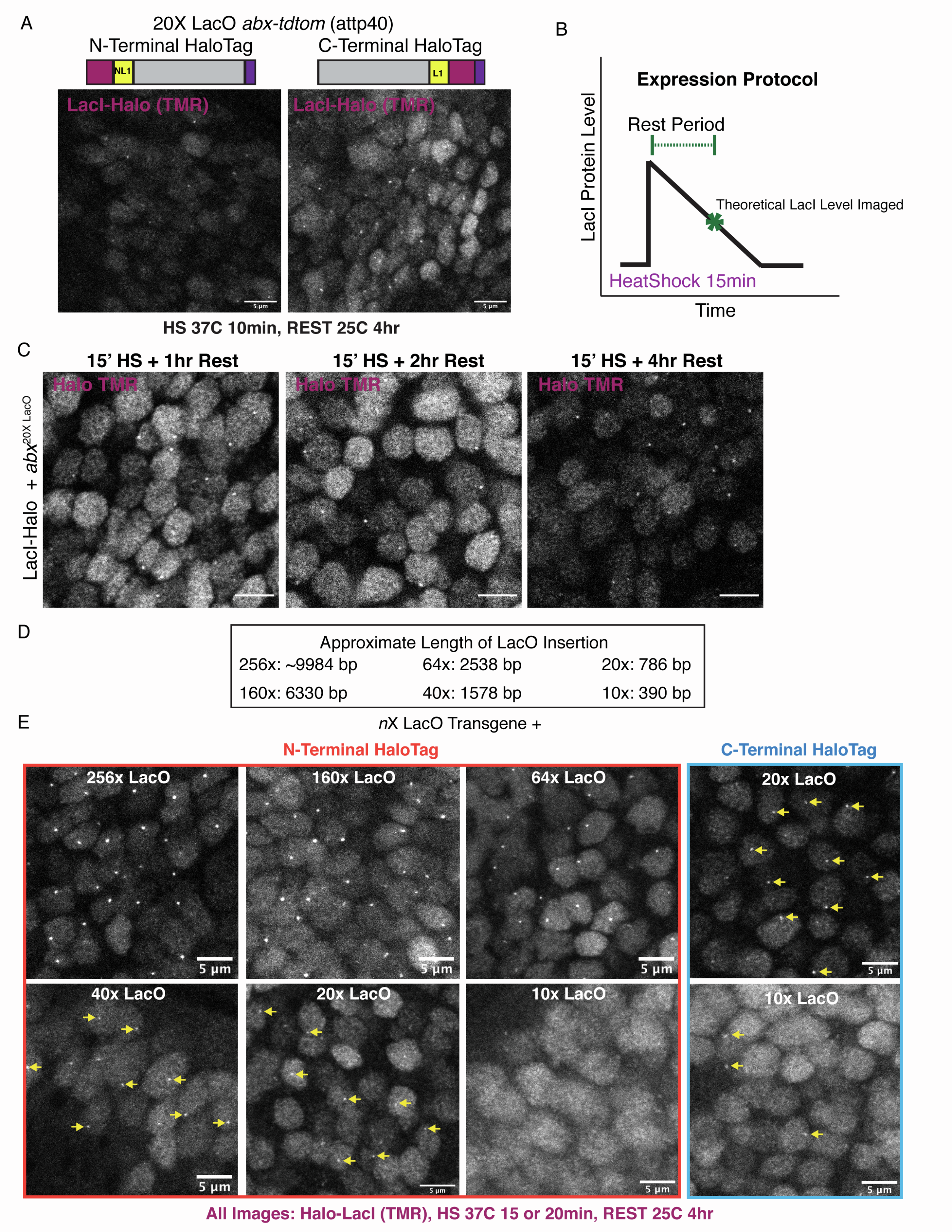
A
20X LacO abx-tdtom (attp40)
N-Terminal HaloTag
C-Terminal HaloTag
NL1
L1
LacI-Halo (TMR)
LacI-Halo (TMR)
5 µm
5 µm
HS 37C 10min, REST 25C 4hr
B
Expression Protocol
LacI Protein Level
Rest Period
Theoretical LacI Level Imaged
HeatShock 15min
Time
C
15' HS + 1hr Rest
15' HS + 2hr Rest
15' HS + 4hr Rest
Halo TMR
Halo TMR
Halo TMR
LacI-Halo + abx20X LacO
D
Approximate Length of LacO Insertion
256x: ~9984 bp
64x: 2538 bp
20x: 786 bp
160x: 6330 bp
40x: 1578 bp
10x: 390 bp
E
nX LacO Transgene +
N-Terminal HaloTag
C-Terminal HaloTag
256x LacO
160x LacO
64x LacO
20x LacO
40x LacO
20x LacO
10x LacO
10x LacO
5 µm
All Images: Halo-LacI (TMR), HS 37C 15 or 20min, REST 25C 4hr

Supplemental Figure 2. Optimization of LacI Expression. Related to Figure 2.

(A) A comparison of a LacI-HaloTag fusion tagged at the N-terminus (left) or C-terminus (right). A colored schematic of each fusion protein depicts domains. The linker (NL1 or L1) is shown in yellow; the NLS in purple; and the HaloTag in magenta. Sequences of the linker and NLS are shown in Figure 2. C-terminal LacI-HaloTag exhibits a greater labeling efficiency than N-terminal LacI-HaloTag.
(B) A schematic of the expression protocol used for FP expression. A 15min HS at 37C is used to induce FP expression. A REST period is used to allow protein turnover in order to reduce background FP signal relative to FP signal at the ROI.
(C) Optimization of the length of REST period for expression protocol. A 1hr, 2hr, and 4hr REST period were tested after a 15min HS. A 4hr REST showed the greatest SNR, and was used for all experiments in the main figures.
(D) Length of LacO tracts tested.
(E) (Left, in orange) N-terminal LacI-HaloTag labeling of a LacO transgene. The 256x LacO construct, not constructed by us, is inserted into Chr III. The remaining LacO constructs (160x, 64x, 40x, 20x, 10x) consisted of a LacO tract upstream of a minimal *abx* CRM (*abxN* (Delker et al., 2019)) and fluorescent reporter inserted into attP40 on Chr II. (Right, in blue) C-terminal LacI-HaloTag labeling of 20X and 10X LacO containing *abx* transgenes. LacI-HaloTag is stained with HaloLigand TMR.

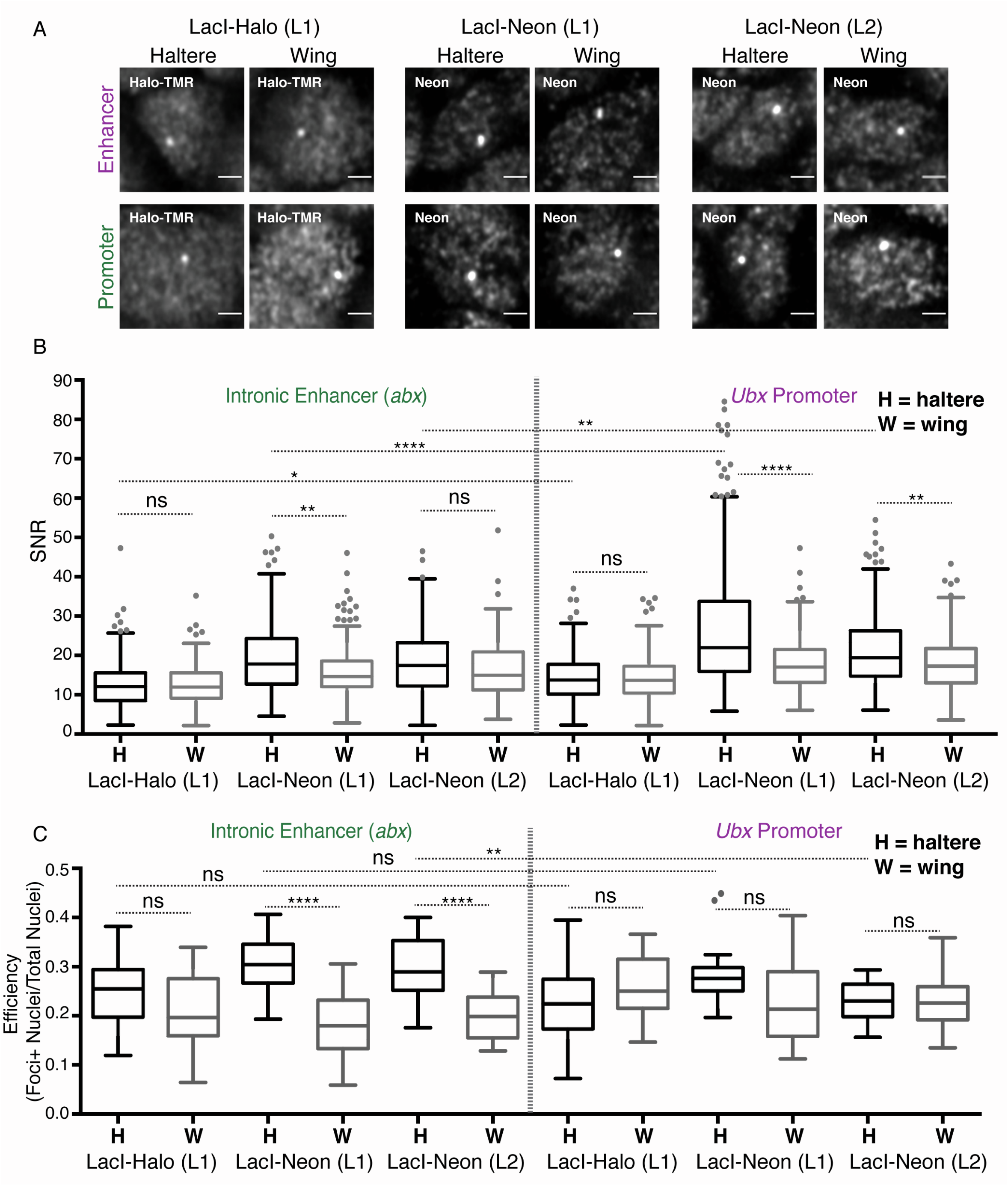

Supplemental Figure 3. Comparison of LacI FP Fusions. Related to Figure 2.

(A) Example images of each LacI FP at the *abx* Enhancer and *Ubx* Promoter in haltere and wing discs. HaloTag fusions are stained with HaloLigand TMR. Native fluorescence is captured from Neon fusions. Scale bars are 1 micron.
(B) SNR of each LacI FP fusion at each ROI in each tissue.
(C) Labeling efficiency of each LacI FP fusion at each ROI in each tissue. Tukey boxplots are used for all graphs. Significance for B and C was tested using a one-way ANOVA analysis followed by Tukey's multiple comparisons test with alpha =.05 in Prism Graphpad (ns = not significant, * $p < .05$, ** $p < .01$, *** $p < .001$, **** $p < .0001$).

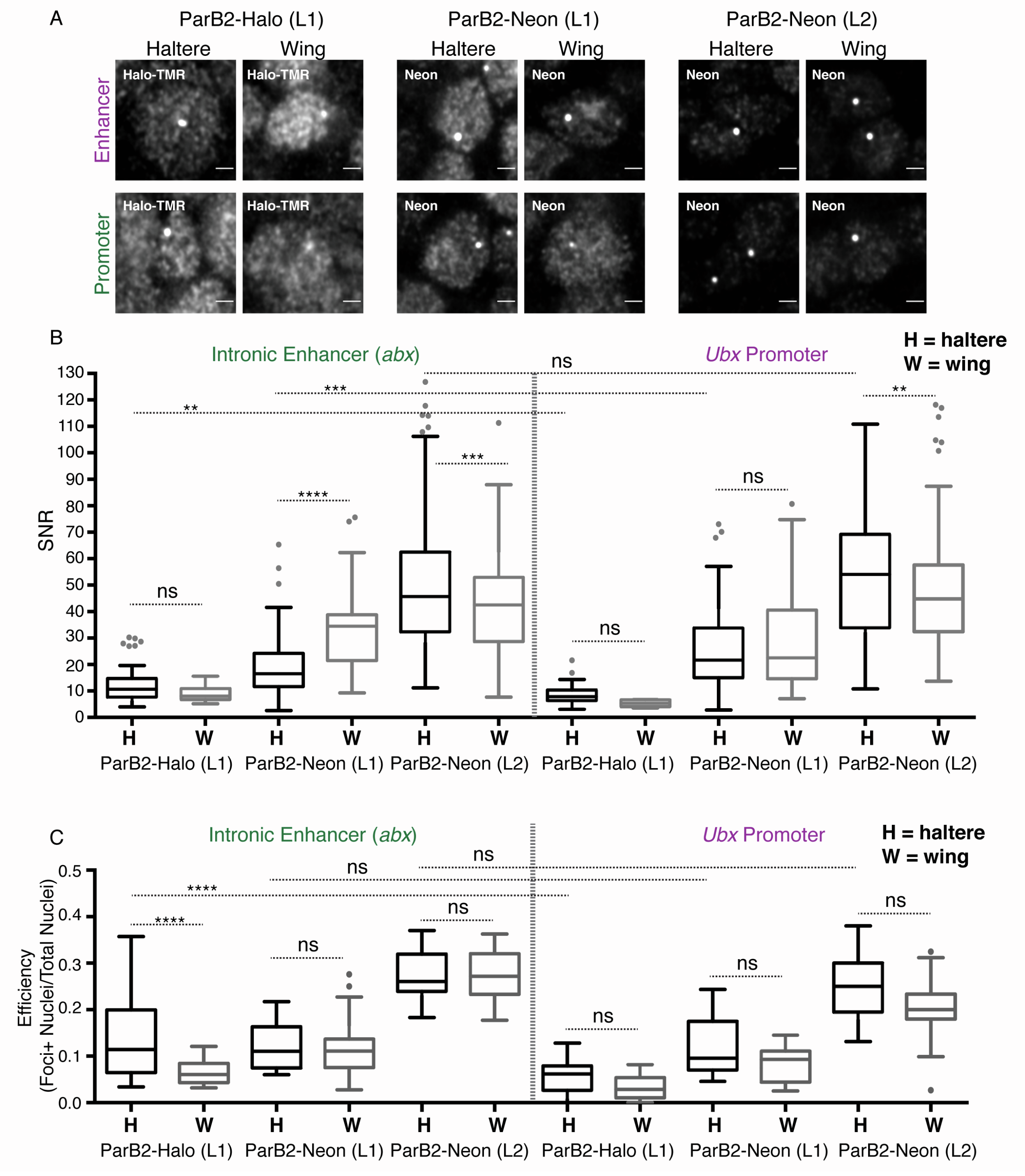

Supplemental Figure 4. Comparison of ParB2 FP Fusions. Related to Figure 3.

(A) Example images of each ParB2 FP at the *abx* Enhancer and *Ubx* Promoter in haltere and wing discs. HaloTag fusions are stained with HaloLigand TMR. Native fluorescence is captured from Neon fusions. Scale bars are 1 micron.
(B) SNR of each ParB2 FP fusion at each ROI in each tissue.
(C) Labeling efficiency of each ParB2 FP fusion at each ROI in each tissue. Tukey boxplots are used for all graphs. All Significance for B and C was tested using a one-way ANOVA analysis followed by Tukey's multiple comparisons test with alpha =.05 in Prism Graphpad (ns = not significant, * $p < .05$, ** $p < .01$, *** $p < .001$, **** $p < .0001$).

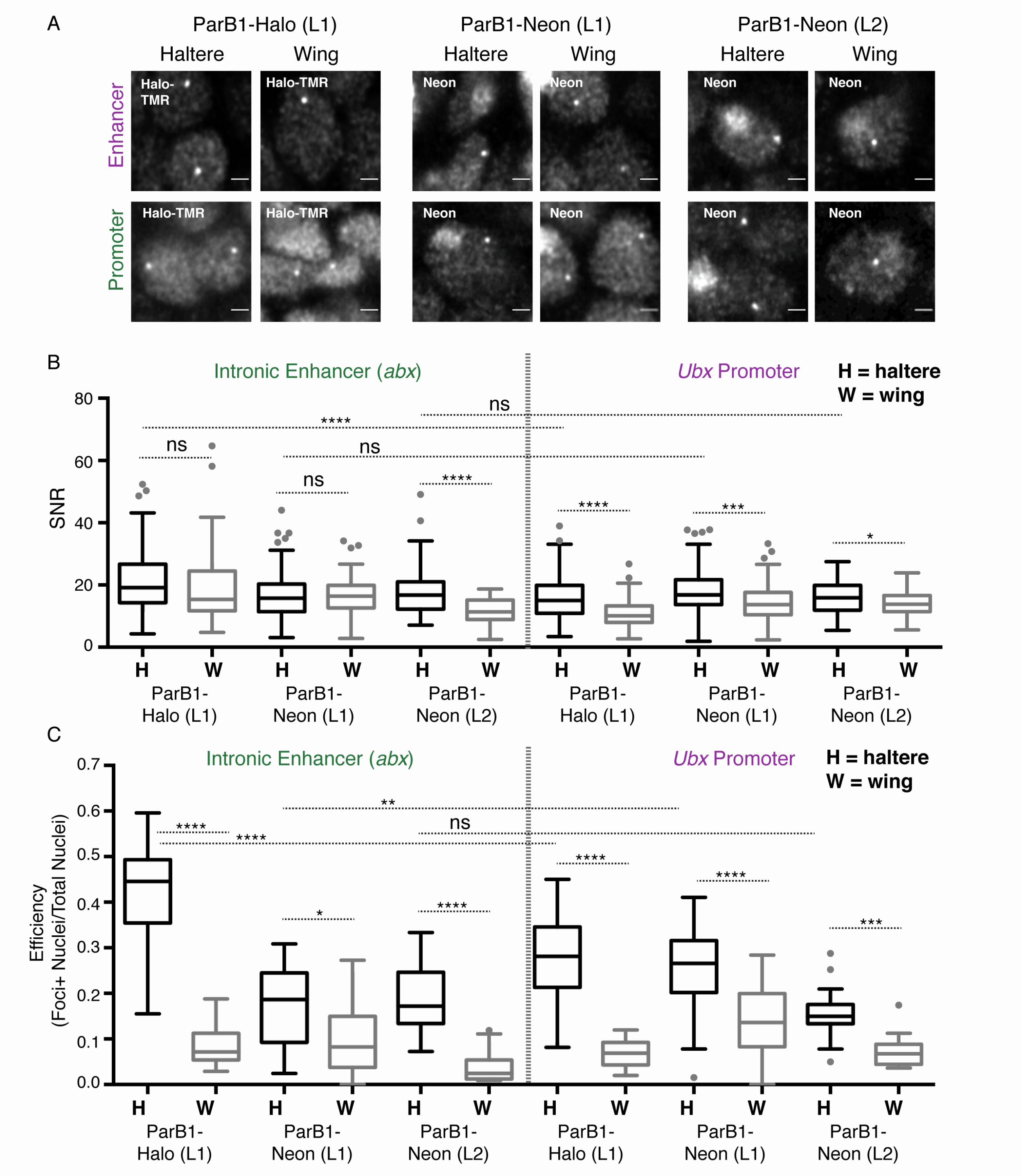

Supplemental Figure 5. Comparison of ParB1 FP Fusions. Related to Figure 4.

(A) Example images of each ParB1 FP at the *abx* Enhancer and *Ubx* Promoter in haltere and wing discs. HaloTag fusions are stained with HaloLigand TMR. Native fluorescence is captured from Neon fusions. Scale bars are 1 micron.
(B) SNR of each ParB1 FP fusion at each ROI in each tissue.
(C) Labeling efficiency of each ParB1 FP fusion at each ROI in each tissue. Tukey boxplots are used for all graphs. Significance for B and C was tested using a one-way ANOVA analysis followed by Tukey's multiple comparisons test with alpha =.05 in Prism Graphpad (ns = not significant, * $p < .05$, ** $p < .01$, *** $p < .001$, **** $p < .0001$).

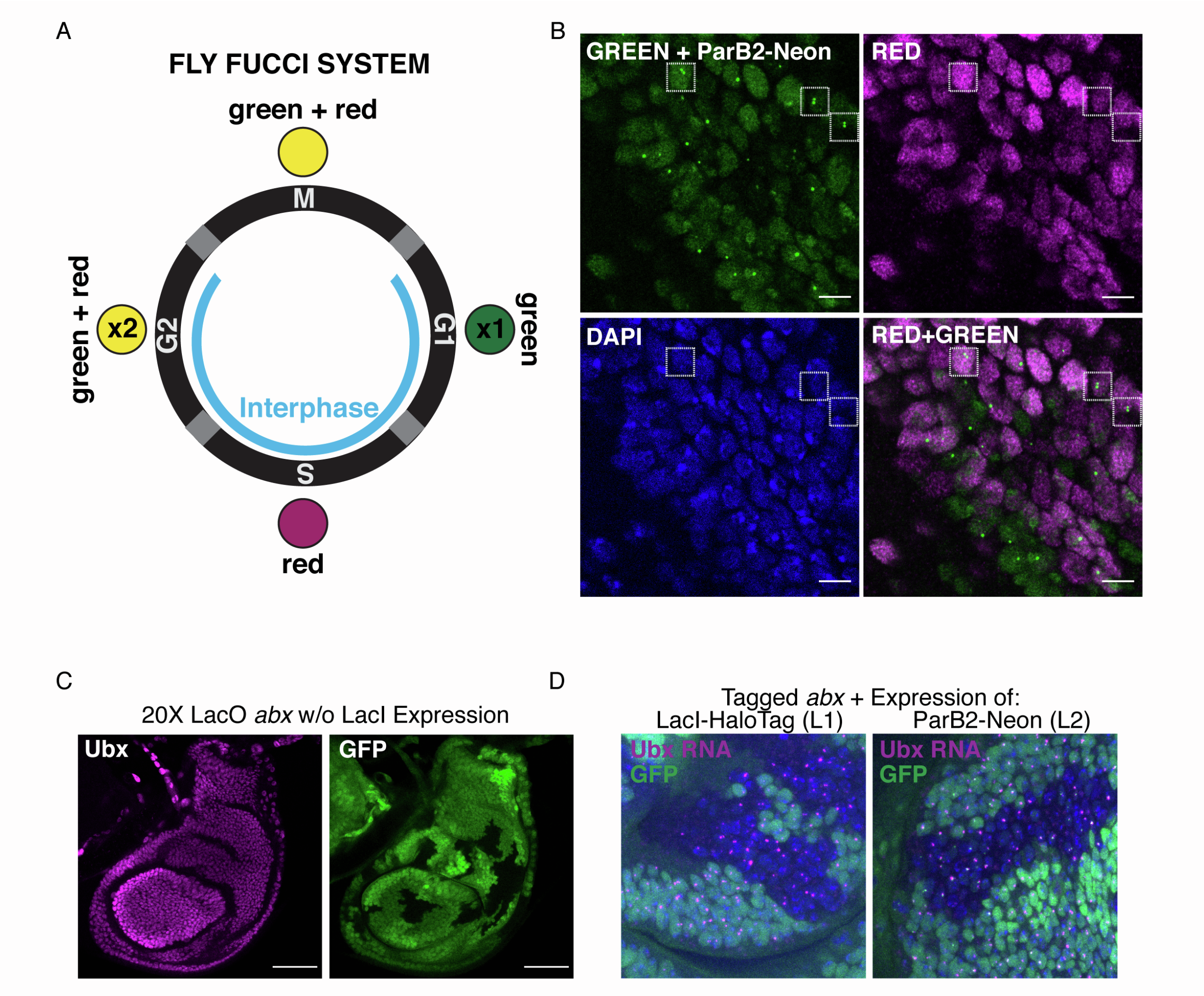

Supplemental Figure 6. Characterization of FP Labeling Systems. Related to Figure 5.

(A) Schematic of the Fly FUCCI system from (Zielke et al., 2014). The stages of interphase can be distinguished by the stabilization of fluorescent degron fusions. G1 is marked by green fluorescence, S is marked with red fluorescence, and G2 with red and green fluorescence. See Materials and Methods for more details.
(B) Haltere Disc image labeling *abx*-ParS2 with ParB2 Neon(L2) in the context of the Fly FUCCI system. Importantly, the green signal comes from both the green FUCCI degron and the ParB2-Neon(L2). Thus, we cannot use green to distinguish stages of Interphase. Instead, we use red fluorescence (shown in magenta) to label nuclei either in S or G2/M phase to find nuclei that could contain 2 copies of the genome. Scale bar is 5 microns.
(C) Mitotic clonal analysis of a 20X LacO tagged *abx* allele in the absence of LacI expression. A Ubx immunostain (magenta) and GFP native fluorescence (green) are shown. Scale bars are 50 microns. In total, 22 clones were observed and all 22 clones showed normal Ubx expression.
(D) Mitotic clonal analysis of tagged *abx* with the expression of either LacI-HaloTag (L1) (left) or ParB2-Neon (L2) (right). smFISH against *Ubx* intron 1 was used to detect nascent *Ubx* transcripts. GFP- clones are homozygous for the tagged *abx* allele. 1 copy of the FP fusion is expressed in all cells. 6 Clones of LacI-HaloTag (L1) were observed and none showed any obvious defect in *Ubx* FISH signal. 2 Clones of ParB2-Neon (L2) were observed and neither showed an obvious defect in *Ubx* FISH signal.
